# The enzymes OSC1 and CYP716A263 produce a high variety of triterpenoids in the latex of *Taraxacum koksaghyz*

**DOI:** 10.1038/s41598-019-42381-w

**Published:** 2019-04-11

**Authors:** Katharina M. Pütter, Nicole van Deenen, Boje Müller, Lea Fuchs, Kirsten Vorwerk, Kristina Unland, Jan Niklas Bröker, Emely Scherer, Claudia Huber, Wolfgang Eisenreich, Dirk Prüfer, Christian Schulze Gronover

**Affiliations:** 1 University of Muenster, Institute of Plant Biology and Biotechnology, Schlossplatz 8, 48143 Muenster, Germany; 2 Fraunhofer Institute for Molecular Biology and Applied Ecology IME, Schlossplatz 8, 48143 Muenster, Germany; 3 Technische Universität München, Chair of Biochemistry, Lichtenbergstraße 4, 85747 Garching, Germany

## Abstract

Only very little is known about the resin composition of natural rubber from the dandelion species *Taraxacum koksaghyz*, thus its full characterization could provide new insights into how the isoprenoid end-products influence the physical properties of natural rubber, and this resin might be a good source of highly diverse triterpenoids. Here, we present a comprehensive analysis of the triterpenoid composition in an acetone extract and identified 13 triterpenes and triterpenoids also including the so far unknown pentacyclic compounds lup-19(21)-en-3-ol (**1**) and its ketone lup-19(21)-en-3-one (**2**). We purified single triterpenes from the acetone extract by developing a two-step HPLC system that is adapted to the structural differences of the described triterpenoids. Furthermore, we isolated six different oxidosqualene cyclases (OSCs) and two P450 enzymes, and we functionally characterized TkOSC1 and CYP716A263 in *Nicotiana benthamiana* and *Saccharomyces cerevisiae* in detail. TkOSC1 is a multifunctional OSC that was capable of synthesizing at least four of the latex-predominant pentacyclic triterpenes (taraxasterol, α-, β-amyrin and lup-19(21)-en-3-ol) while CYP716A263 oxidized pentacyclic triterpenes at the C-3 position. The identified enzymes responsible for biosynthesis and modification of pentacyclic triterpenes in *T. koksaghyz* latex may represent excellent tools for bioengineering approaches to produce pentacyclic triterpenes heterologously.

## Introduction

Over 20,000 angiosperm species produce latex, a highly specialized cytoplasm, in tubular cells called laticifers^1^. Latex can serve as a storage tissue for secondary metabolites in very high concentrations, and it may also help defend against herbivorous insects^2,3^. The dandelion species *Taraxacum koksaghyz* produces a plethora of secondary metabolites in its latex, and this plant has been thoroughly investigated because its latex tissue contains significant levels of high quality poly(*cis*-1,4-isoprene), the main component of natural rubber (NR)^4–6^. Additionally, the latex of *Taraxacum* species also contains substantial amounts of pentacyclic triterpenes^7^. These pentacyclic triterpenes exhibit extraordinary biological activities against fungi and bacteria, making them highly attractive for agricultural and pharmaceutical applications^8^. Furthermore, these compounds have been described to be one of the components of natural rubber, besides fatty acids and proteins, that might influence the physical properties of the polymer^9^.

Although the pentacyclic triterpenes in *Taraxacum* species are of significant interest, they have not yet been fully characterized. Thus, it is pivotal to elucidate the detailed pentacyclic triterpene composition of *T. koksaghyz* NR. Accordingly, a reliable method needed to be established to purify single triterpenes and triterpenoids. Moreover, as overall amounts *in planta* often prove economically nonviable, profound knowledge about triterpene-synthesizing and -modifying enzymes might help to establish bioengineering approaches for the production of pentacyclic triterpenoids and NR with altered properties.

The pentacyclic triterpenes, 30-carbon compounds, are the largest class of triterpenes and derive from the cytosolic mevalonate pathway. They are synthesized from isopentenyl diphosphate (IPP) generated by the mevalonate pathway and its isomer, dimethylallyl diphosphate (DMAPP). Successive condensation reactions catalysed by farnesyl diphosphate synthase and squalene synthase lead to the biosynthesis of squalene, a linear 30-carbon compound, which subsequently can be oxidized by squalene epoxidase to 2,3-oxidosqualene, the precursor for triterpene biosynthesis.

Once 2,3-oxidosqualene is available, the formation of pentacyclic triterpenes requires a cyclization reaction. The cyclization reaction is catalysed by oxidosqualene cyclases (OSCs) and represents one of the most complex enzymatic reactions in terpene metabolism^10^. Until now, over 20,000 disparate triterpenes belonging to 100 different scaffolds have been identified as products of OSCs^11^. Here, cyclization of the chair-boat-chair conformation of 2,3-oxidosqualene yields the tetracyclic protosteryl cation intermediate that generates sterols, whereas cyclization of the chair-chair-chair conformation of 2,3-oxidosqualene produces the tetracyclic dammarenyl cation that can undergo subsequent ring expansion to form pentacyclic triterpenes. These can further be modified by cytochrome P450 monooxygenases (P450s) or glycosyltransferases, yielding triterpenoids and saponins, respectively.

So far, enzymes involved in the biosynthesis and modification of pentacyclic triterpenes and triterpenoids have been identified in several plant species, including the multifunctional lupeol synthase TkLUP from *T. koksaghyz*^12–15^. Regarding P450s, studies have predominantly identified the CYP716 family members as responsible for oxidizing pentacyclic triterpenes, most commonly at the C-28 position^16^.

Here, we performed a detailed analysis of the pentacyclic triterpenoid composition in acetone extracts of natural rubber from *T. koksaghyz* roots. An HPLC-based method for the purification of single triterpenoids was developed and used for the identification of a new pentacyclic triterpene, lup-19(21)-en-3-ol, with its corresponding pentacyclic triterpenoid, the ketone lup-19(21)-en-3-one. In addition, the *T. koksaghyz* multifunctional OSC enzyme TkOSC1 and the scaffold-modifying P450 enzyme CYP716A263 that catalyses the formation of several triterpenoids in latex were functionally characterized for the first time.

## Results

### *Taraxacum koksaghyz* natural rubber acetone extract reveals triterpene composition

**Figure 1:**
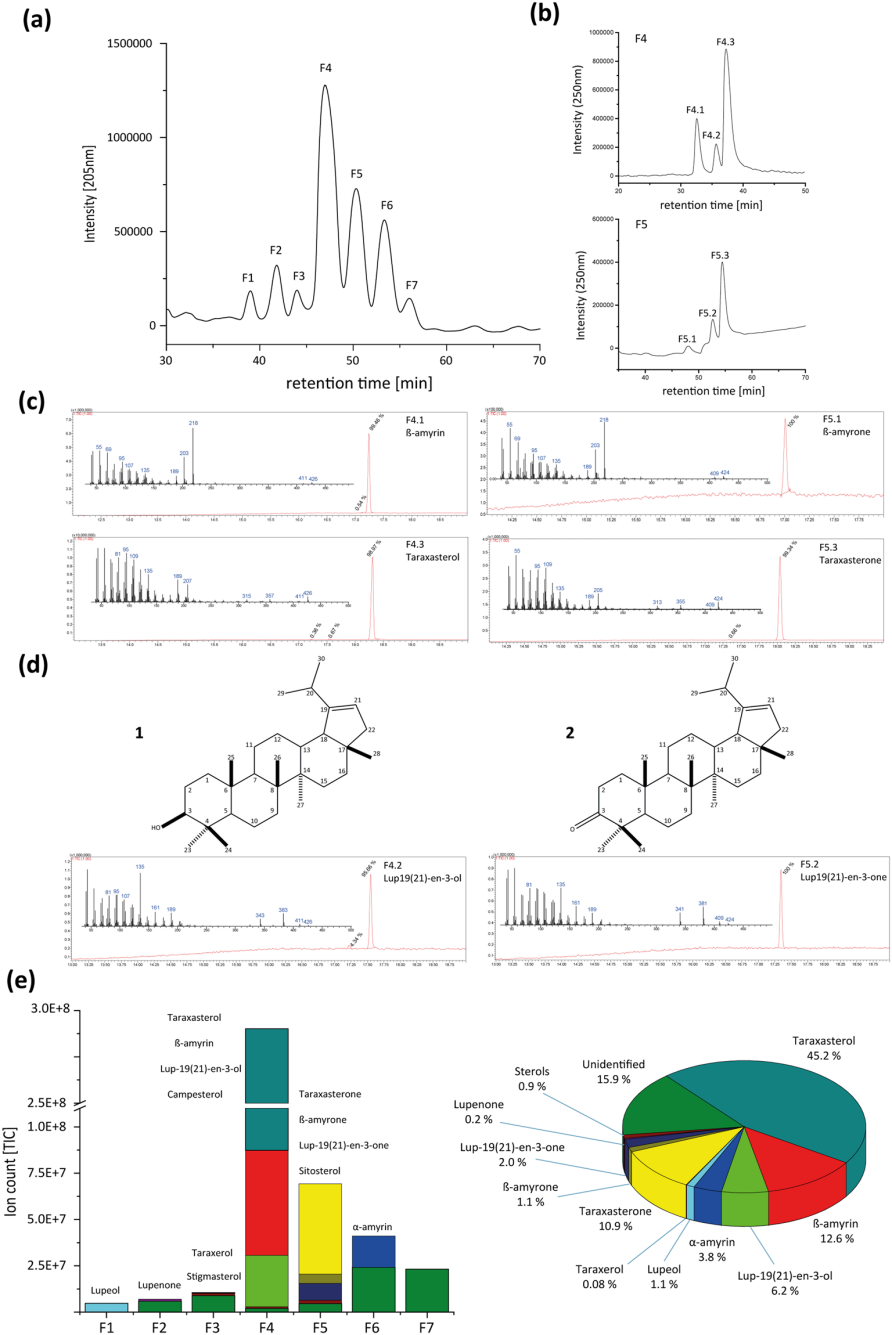
Triterpene purification by HPLC. Single triterpenes were separated using (**a**) an Ultra C18 column followed by (**b**) an Ultra Biphenyl column. (**c**) GC-MS spectra of β-amyrin and taraxasterol and their ketone derivatives purified from fraction 4 (F4) and 5 (F5), respectively. (**d**) Molecular structure and GC-MS spectra of the newly identified lup-19(21)-en-3-ol and its ketone derivative lup-19(21)-en-3-one purified from F4 and F5, respectively. (**e**) Quantification of triterpenes in single C18-HPLC fractions as a sum of all fractions.

Besides the main component poly(*cis*-1,4-isoprene), natural rubber contains additional substances like proteins, fatty acids and triterpenes that influence the physical properties of the polymer^9^. To gain a detailed overview about the single triterpenes that play a role in natural rubber characteristics, a lipid fraction was extracted from *T. koksaghyz* NR using acetone as a solvent. The acetone extract was separated on a C18 column by HPLC and seven main fractions could be observed using UV detection at 205 nm (Fig. 1a). They were collected and subsequently analysed by GC-MS (Supplementary Fig. S1). Only fraction (F) 1 contained one single triterpene that could be identified as lupeol. Fractions F2-7 comprised a mixture of substances containing two to five different C30 compounds. Utilizing an additional separation step by HPLC with another stationary phase (biphenyl column), we were able to further separate the single triterpenes from each other, as shown, for example, for F4 and F5 (Fig. 1b). Subsequent GC-MS analysis showed that we successfully isolated the substances with a high purity grade varying from 98.97% up to 100% (Fig. 1c). We detected different pentacyclic triterpenes that are described to be highly abundant in the roots of *Taraxacum koksaghyz*^17^ in four of the seven C18 fractions; these pentacyclic triterpenes included taraxasterol and β-amyrin in fraction F4 (Fig. 1c), α-amyrin in F6, lupeol in F1 and taraxerol in F3 (Supplementary Fig. S1). In addition to the alcohols, we also identified ketone derivatives of taraxasterol, β-amyrin and lupeol in two fractions, namely taraxasterone and β-amyrone in F5 (Fig. 1c) and lupenone in F2 (Supplementary Fig. S1). Furthermore, sterol compounds that have been previously described to be present in root material of *Taraxacum koksaghyz*^17^ were detected in three fractions: stigmasterol (F3), campesterol (F4) and sitosterol (F5) (Supplementary Fig. S1).

Moreover, after several rounds of repeating the two-step HPLC runs, we were able to isolate mg amounts of single triterpenes for subsequent structural analysis by NMR. Using this system, a third so far unknown triterpene appeared in F4 and its corresponding ketone was detected in F5. About 1.5 mg of the triterpene with a purity grade of 95.66% and 0.3 mg of pure substance (100%) of the corresponding ketone was purified for NMR analysis, resulting in the identification of an as-yet unknown pentacyclic triterpene, lup-19(21)-en-3-ol (**1**), and its corresponding pentacyclic triterpenoid, the ketone lup-19(21)-en-3-one (**2**) (Fig. 1d, NMR data in Supplementary Table S1).

All triterpenes and triterpenoids that were identified in the acetone extract are summarized in Fig. 1e. The peak areas of the GC-MS total ions were used to quantify the relative abundance of the single triterpenes in the C18-fractions and to calculate the percentage amount of all detected triterpenes in the lipid fraction. The most abundant pentacyclic triterpene in the lipid fraction was taraxasterol (about 45.2%), followed by β-amyrin (12.6%), lup-19(21)-en-3-ol (6.2%), α-amyrin (3.8%), lupeol (1.1%) and taraxerol (0.08%). The corresponding ketone derivatives comprise 14.2% of the total lipid extracts. Only a low percentage of the total triterpene amount (0.9%) represented sterols. In all fractions, further compounds could be detected and classified as triterpenes due to their GC-MS profiles (Supplementary Fig. S1), but the detailed molecular structure of those compounds is still unknown. These as-yet unidentified triterpenes represent about 15.9% of the total lipid extract. Interestingly, more than 14% of the successfully identified triterpenes appear as ketones. Therefore, we focused on the enzymes that are responsible for triterpene synthesis as well as for the modification of triterpenes in the latex of *T. koksaghyz*.

### Identification and sequence analysis of *OSC* and *CYP* genes in *T. koksaghyz*

We recently performed a functional characterization of a lupeol synthase from *T. koksaghyz* (TkLUP, GenBank accession number MG646375) that synthesizes not only lupeol but also β-amyrin in minor amounts when heterologously expressed in yeast^15^. To identify further OSC enzymes that synthesize highly abundant triterpene end-products in dandelion latex such as taraxasterol, we searched for *OSC* sequences in *T. koksaghyz* RNA-Seq data including root, latex, leaf and flower material.

Based on comparison with the *Artemisia annua OSC2* (*AaOSC2*, GenBank accession number KF309252; Moses *et al*.^12^), which is involved in triterpene biosynthesis in trichomes, we found three putative *TkOSC* cDNA sequence fragments and three full-length sequences. Corresponding sequences were found in *T. koksaghyz* genome data^18^ and were used to extend all fragments, finally giving six full-length *OSC* open reading frames. Primers derived from those sequences were used to amplify corresponding cDNAs from *T. koksaghyz* latex and root material, resulting in *TkOSC1*-6 comprising 2,277 to 2,313 bp (sequence properties are summarized in Supplementary Table S2).

As the presence of ketones such as taraxasterone and β-amyrone suggest that P450s are involved in the oxidation reactions, we also mined RNA-Seq and genome data for P450 sequences. Accordingly, RNA-Seq analyses carried out with *T. koksaghyz* root, latex and leaf material revealed two contigs of interest comprising P450 open reading frames. One corresponding mRNA was abundant in latex, but less present in root and leaf tissues, and contained a complete ORF with striking similarity (63%) to *A. annua CYP71*6*A14v2* (*AaCYP716A14v2*, GenBank accession number KF309251; Moses *et al*.^12^). Subsequently, a 1,422-bp full-length sequence was amplified from *T. koksaghyz* latex cDNA and annotated as *CYP716A263* by the P450 nomenclature committee (GenBank accession number MG646382). The other putative P450-coding contig is linked to high transcript levels in leaves compared to latex and roots and displayed high similarity to *A. annua CYP716D22* (*AaCYP716D22*, GenBank accession number KF309250; Moses *et al*.^12^). Primers based on the contig sequence served to amplify a 1,440-bp coding sequence from *T. koksaghyz* leaf cDNA and was annotated *CYP716D60* by the P450 nomenclature committee (GenBank accession number MG646383, sequence properties are summarized in Supplementary Table S2, primers for cloning of full-length sequences are given in Supplementary Table S4).

In order to analyse whether these genes encode functional proteins, *in silico* analyses were performed with the amino acid sequences deduced from the full-length cDNAs isolated from *T. koksaghyz* tissues.

**Figure 2:**
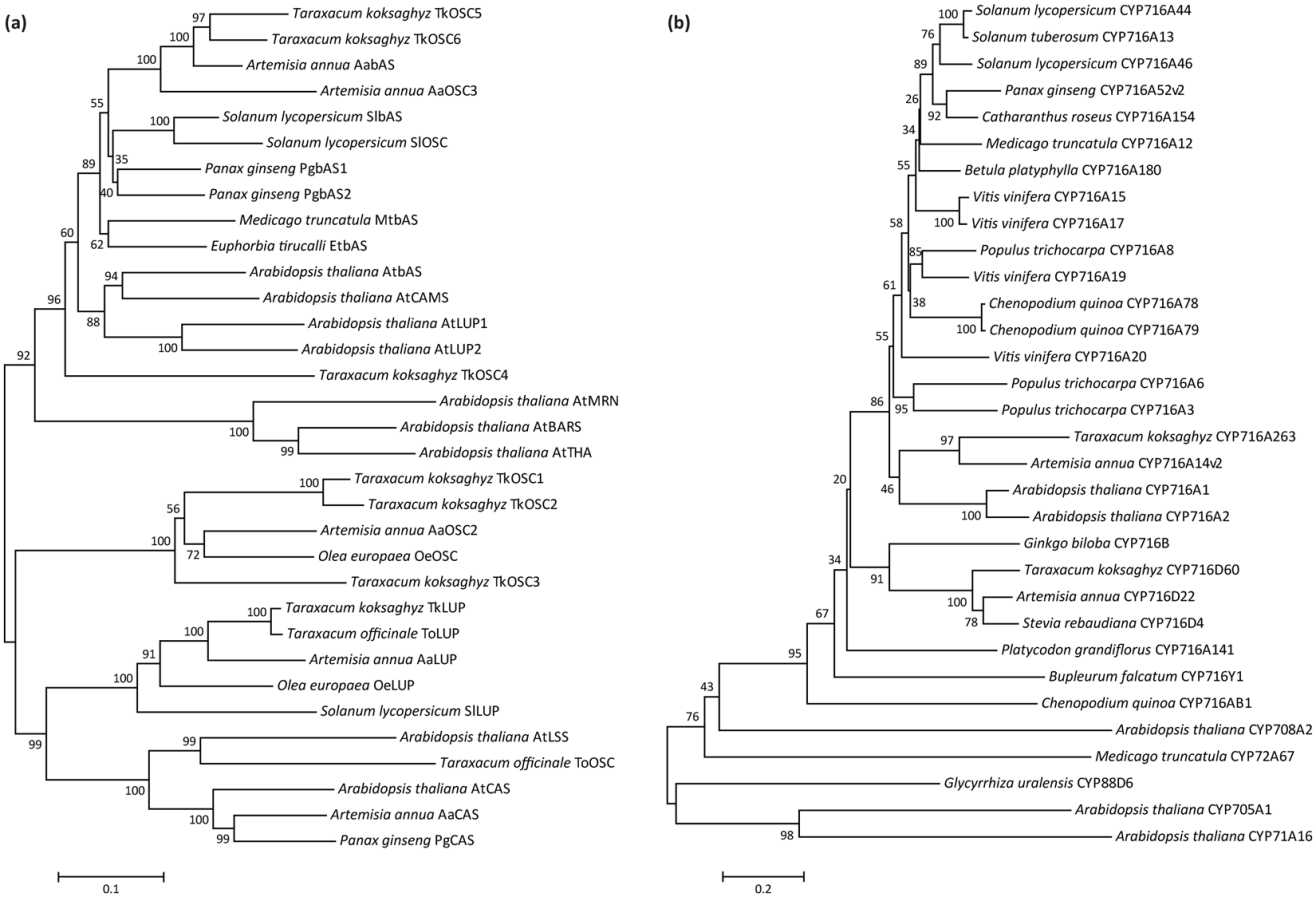
Cladogram of (**a**) OSC and (**b**) P450 amino acid sequences. The cladogram was constructed by MEGA6 software^57^ (http://www.megasoftware.net/), using Neighbour-joining with a bootstrap of 1000 replicates. Protein sequences were obtained from GenBank (https://www.ncbi.nlm.nih.gov/genbank/) and are provided in Supplementary Table S3.

For TkOSC1-6 and TkLUP, deduced polypeptides comprised 758 to 770 amino acids (Supplementary Table S2), whereas TkOSC1 compared to TkOSC2 displayed the highest (94%) sequence identity among the newly identified sequences. Analysis of the deduced amino acid sequence revealed that conserved domains reported for OSCs were present with only slight modifications for TkOSC4-6. Those included a domain essential for product determination, one region involved in substrate binding and polycyclization initiation and six repeated QW motifs involved in stabilization of carbocationic intermediates and localized near the 5′ and 3′ ends of the proteins^10,19–23^ (see Supplementary Fig. S2a). Based on amino acid sequence alignments, a cladogram was established illustrating that the seven sequences derived from *T. kokzaghyz* cluster among different OSC types (Fig. 2a). Thus, TkLUP clusters among other lupeol synthases, whereby highest sequence similarities were observed between TkLUP and ToLUP (98%) as well as between TkLUP and AaLUP (85%). The multifunctional AaOSC2 clustered to TkOSC1 (76%), TkOSC2 (74%) and TkOSC3 (77%), whereas the β-amyrin synthases AabAS and PgbAS1 showed highest sequence similarities to TkOSC4 (70%), TkOSC5 (86%) and TkOSC6 (89%), respectively.

Concerning P450 sequences, *CYP716A263* and *CYP716D60* encoded for polypeptides of 473 and 479 amino acids, respectively, and showed a sequence identity of 44%. Subsequent sequence analysis revealed that both P450 sequences isolated from *T. koksaghyz* possessed the conserved domains characteristic for P450s. These included a hydrophobic region at the N-terminus necessary for anchoring the enzyme to the membrane and a proline-rich region next to it, the I-helix involved in oxygen binding, and the E-R-R triad essential for catalysis and stabilizing the core structure, and the heme-binding motif near the C-terminus^14,24^ (see Supplementary Fig. S2b). Thus, it can be concluded that both P450s might represent functional members of the P450 superfamily. Next, a cladogram was calculated and served to depict the relatedness of different P450 members with the isolated sequences (Fig. 2b). CYP716A263 and CYP716D60 both cluster within their corresponding subfamilies among CYP716 family members. For CYP716A263, the highest sequence identity was reached for CYP716A14v2 from *A. annua* (63%), while CYP716D60 exhibited a sequence identity of 79% compared to *A. annua* CYP716D22.

### Spatial expression patterns of *OSCs* and *P450s*

In order to assess the spatial expression patterns of all *TkOSC*, *TkLUP* and both *TkCYP* genes investigated, qRT-PCR was performed with cDNA from latex, root, leaf, peduncle and flower of 12-week-old *T. koksaghyz* plants grown under greenhouse conditions.

**Figure 3:**
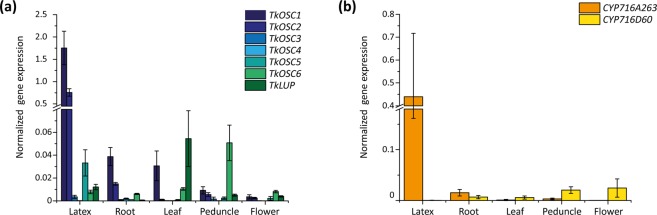
Spatial *TkOSC1*-*6*, *TkLUP*, *CYP716A263* and *CYP716D6* mRNA expression profile in wild-type *T. koksaghyz* plants determined by qRT-PCR. The corresponding mRNA levels were normalized against the constitutive genes elongation factor 1 α (*TkEF1α*) and ribosomal protein L27 (*TkRP*) from *T. koksaghyz*. Bars represent standard errors of nine independent wild-type plants. (**a**) *TkOSC1*-6 and *TkLUP* and (**b**) *CYP716A263* and *CYP716D60* mRNA levels in latex, roots, leaves, peduncles and flowers of 12-week-old wild-type *T. koksaghyz* plants. Primer sequences can be obtained from Supplementary Table S4, and qRT-PCR primer efficiencies are summarized in Supplementary Table S5.

The qRT-PCR revealed that *TkOSC1, TkOSC2* and *TkOSC5* were the three *OSC* genes with the highest expression in latex tissue. In leaf, *TkOSC1* and *TkLUP* were the most abundant transcripts, while *TkOSC6* exhibited the highest expression in peduncle and flower. All other *OSC* transcripts were only present in minor amounts (Fig. 3a). As our goal was to identify enzymes synthesizing pentacyclic triterpenes in dandelion latex, *TkOSC1*, *TkOSC2* and *TkOSC5* were the focus of our further studies. Moreover, we also characterized *TkOSC6* as it exhibited a spatially distinct expression pattern with its highest expression in peduncle while being closely related to TkOSC5 (88%).

As RNA-Seq analyses for P450s have already revealed, *CYP716A263* was highly expressed in latex tissue, more than 10-fold compared to in roots, with only minor amounts of *CYP716A263* transcripts present in leaf, peduncle and flower material. Contrarily, *CYP716D60* expression was highest in peduncle and flower, with low expression levels in root, leaf and latex (Fig. 3b). Although both P450 genes revealed tissue-specific expression, CYP716A263 and CYP716D60 were analysed regarding their functionality in the heterologous expression system *Nicotiana benthamiana*.

### Combinatorial heterologous expression of *OSCs* and *P450s* in *Nicotiana benthamiana* and *Saccharomyces cerevisiae*

In order to determine the functionality of the isolated OSCs and P450s, heterologous expression was carried out in tobacco (*N. benthamiana*). The full-length cDNAs of the genes of interest (*TkOSC1, TkOSC2, TkOSC5, TkOSC6, CYP716A263, CYP716D60*) were cloned into plant transformation vectors and transformed into *Agrobacterium tumefaciens* strains suitable for transient expression in tobacco. Subsequently, tobacco leaves were infiltrated with *Agrobacterium* strains harbouring the corresponding vectors. In order to increase isoprenoids, especially the triterpenoid end-products, the flux through the mevalonate pathway had to be increased substantially. This was achieved by simultaneous overexpression of a truncated form of the rate-limiting enzyme 3-hydroxy-3-methylglutaryl-CoA reductase 1 (HMGR1) from *T. koksaghyz* that contained only the catalytic domain (*Tkhmgrc1*). Overexpression of *Tbhmgrc1* from the closely related dandelion species *T. brevicorniculatum* in *N. benthamiana* leaves has already been shown to result in a considerable increase in mevalonate pathway-derived isoprenoid end-products^25^. Triterpenes and triterpenoids were identified by comparing the retention times (Rts) of standards with the corresponding peaks observed in GC-MS chromatograms (selected ion monitoring (SIM) with m/z 95 and 218 was used for specific detection of pentacyclic triterpenes).

**Figure 4:**
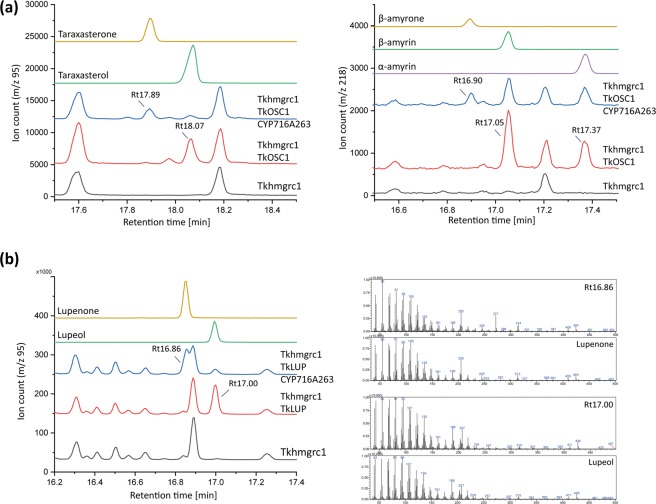
Heterologous expression of *T. koksaghyz* (**a**) *TkOSC1* and (**b**) *TkLUP* with *Tkhmgrc1* and *CYP716A263* in *N. benthamiana*. Selected ion monitoring (SIM) GC-chromatograms of leaf extracts from infiltrated tobacco plants (left) and mass spectra of putative triterpene compounds (right) at specific retention times (Rt).

GC-MS chromatogram analyses of leaf extracts 7 days post infiltration revealed that expression of *Tkhmgrc1* and *TkOSC1* in *N. benthamiana* leaves led to the detection of additional peaks using SIM with m/z 95 and 218 compared to the expression of *Tkhmgrc1* alone (Fig. 4a). The Rts indicated the production of taraxasterol (Rt 18.07 min, m/z 95), α-amyrin and β-amyrin (Rt 17.37 min and Rt 17.05 min, m/z 218). However, due to a low signal-to-noise ratio that is caused by endogenous triterpenoids from the heterologous host *N. benthamiana*, obtaining defined mass spectra for irrevocable identification was not possible. The additional expression of *CYP716A263* resulted in the detection of two additional peaks that might represent taraxasterone (Rt 17.89 min, m/z 95) and β-amyrone (Rt 16.90 min, m/z 218) due to the Rts of the corresponding standards (Fig. 4a).

In order to prove that CYP716A263 is able to modify further pentacyclic triterpene end-products, additional infiltration experiments were performed with TkLUP. GC-MS chromatogram analyses of leaf extracts 7 days post infiltration revealed that expression of *TkLUP* in combination with *Tkhmgrc1* led to the predominant production of lupeol, which was identified using SIM with m/z 95 (Fig. 4b, Rt 17.00 min), while the synthesis of β-amyrin by TkLUP could not be detected in crude leaf extracts of *N. benthamiana* (Supplementary Fig. S3a). In a next step, *Tkhmgrc1*, *TkLUP* and *CYP716A263* were expressed simultaneously. GC-MS analysis of *N. benthamiana* leaf extracts revealed peaks corresponding to diminished levels of lupeol compared to the previous infiltration, but also the presence of masses and an Rt matching its ketone, lupenone (Fig. 4b, Rt 16.86 min). Thus, CYP716A263 is capable of converting lupeol into lupenone and exhibits a C-3 oxidation activity. Furthermore, the infiltration experiments indicate that TkOSC1 is able to produce taraxasterol, α-amyrin and β-amyrin, and that the additional co-expression of CYP716A263 resulted in the production of the corresponding ketones. Therefore, heterologous expression in *N. benthamiana* proved the functionality of TkOSC1 and CYP716A263 even though the detailed identification of all single triterpenoid products was not possible using crude leaf extracts.

Additionally, *TkOSC6* was co-expressed with *Tkhmgrc1* in *N. benthamiana*. Here, GC-MS chromatogram analyses indicated the predominant production of β-amyrin (Rt 17.47, m/z 218, Supplementary Fig. 4a), next to minor amounts of α-amyrin (Rt 17.82, m/z 218, Supplementary Fig. 4a) and an additional unidentified compound (Rt 18.92, m/z 218, Supplementary Fig. 4a). Furthermore, the expression of *Tkhmgrc1*, *TkOSC6* and *CYP716A263* in *N. benthamiana* confirmed the presence of β-amyrone (Rt 17.31; m/z 218, Supplementary Fig. 4a) and therefore, the C3-oxidation activity of *CYP716A263*. Contrarily, the coexpression of *Tkhmgrc1*, *TkOSC2* and *TkOSC5* as well as the coexpression of *Tkhmgrc1*, *TkLUP/TkOSC1* and *CYP716D60* did not result in the occurrence of additional peaks that might represent triterpenoid compounds (Supplementary Fig. S3b–d). Consequently, TkOSC2, TkOSC5 and CYP716D60 were not considered in further studies.

In order to confirm the functionality of the enzymes TkOSC1, TkOSC6 and CYP716A263, and to further identify the products of TkOSC1/CYP716A263 and TkOSC6/CYP716A263 in more detail, *S. cerevisiae* cells were utilized as an additional heterologous expression system that does not produce pentacyclic triterpenoids which would interfere with GC-MS detection. Here, we used an engineered CEN.PK2-1C yeast strain (rox1::P_GAL1_-tHMGR P_GAL10_-ERG13; P_ERG7_Δ::P_CTR3_) that employed several stably transformed modifications leading to an enhanced flux of precursors of the MVA pathway towards triterpene biosynthesis with a concomitant inducible suppression of sterol biosynthesis^15^. *TkOSC1* and *TkOSC6* were expressed under the control of the galactose-inducible promoter GAL1. Initial expression experiments revealed that TkOSC1 was far less active when compared to TkLUP, which might result from a different leader peptide for ER membrane localization. Thus, the TkOSC1 N-terminus (aa 1–30) was replaced by the TkLUP N-terminus (aa 1–21). TkOSC6 showed sufficient activity in the heterologous *S. cerevisiae* system and consequently was not modified. *CYP716A263* was expressed together with *AtR2* (GenBank accession number X66017), a NADPH-cytochrome P450 reductase (CPR), under the control of galactose-inducible promoters, as previous analyses had revealed that yeast-endogenous CPR do not couple well with plant P450 enzymes and that AtR2 is suitable for heterologous expression in yeast^26,27^. Respective yeast strains were cultivated in the presence of CuSO_4_ in order to repress sterol biosynthesis, enzyme expression was induced with galactose, and cells were harvested after they reached a certain density. Subsequently, yeast extracts were analysed via GC-MS.

**Figure 5:**
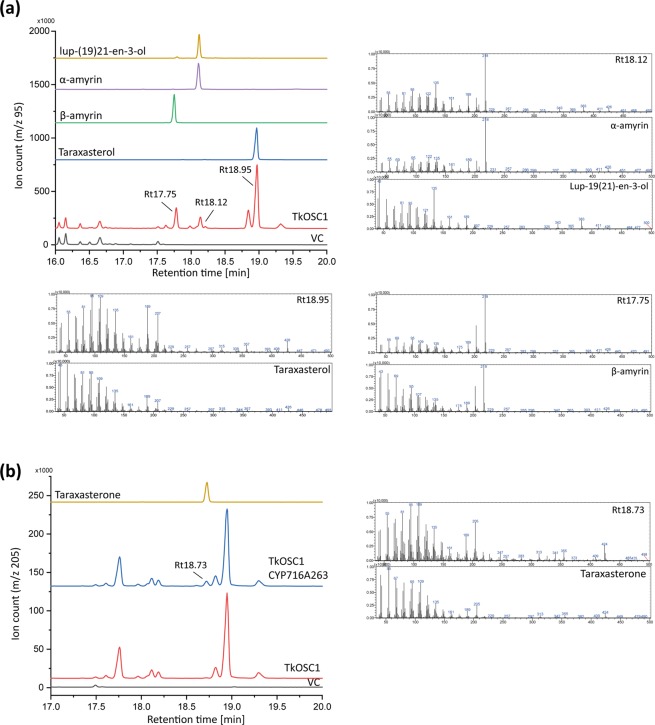
Heterologous expression of *T. koksaghyz* (**a**) *TkOSC1* and (**b**) *TkOSC1* with *CYP716A263* in CEN.PK2-1C yeast strain. Selected ion monitoring (SIM) GC-chromatograms of yeast extracts from pelleted freeze-dried cells (left) and mass spectra of putative triterpene compounds (right) at specific retention times (Rt).

By expressing *TkOSC1* in CEN.PK2-1C yeast, several different peaks were detected in the chromatogram of m/z 95 (Fig. 5a). Two of them were clearly identified as β-amyrin (Rt 17.75 min) and taraxasterol (Rt 18.95 min) by Rt and corresponding mass spectra. At an Rt of 18.12 min, a double peak occurred representing an overlay of the mass signals for α-amyrin and lup-19(21)-en-3-ol. As shown in Supplementary Fig. S5, three of the additional peaks matched to as-yet unknown triterpenes that were also detected in HPLC fractions of the rubber acetone extract (F2 and F5, Supplementary Fig. S1).

Hence, *TkOSC1* encodes for a multifunctional OSC that is able to synthesize at least seven different triterpenes that are present in *T. koksaghyz* latex, including taraxasterol, α-amyrin, β-amyrin and the newly identified lup-19(21)-en-3-ol.

The coexpression of *TkOSC1* and *CYP716A263/AtR2* in yeast resulted in the detection of one additional peak by analysing ion signals at m/z 205 that could be identified as taraxasterone by Rt and mass spectra (Fig. 5b). Therefore, taraxasterol, as the most abundant product, was converted to taraxasterone, as the respective C-3 oxidation product in *TkOSC1/CYP716A263/AtR2*-expressing yeast cells. Similarly, *TkOSC6* expression in yeast led to the identification of β-amyrin as main product (Rt 17.47 min, m/z 218, Supplementary Fig. 4b), which was converted to β-amyrone by coexpression of *TkOSC6* and *CYP716A263/AtR2* (RT 17.31, m/z 218, Supplementary Fig. 4b). Consequently, CYP716A263 C-3 oxidation activity was proven in the heterologous tobacco as well as the yeast expression system by coexpression with different OSCs.

## Discussion

Pentacyclic triterpenes and triterpenoids exhibit an enormous potential for industrial and pharmaceutical applications^8^. However, extraction often proves economically nonviable, as overall amounts *in planta* are low and biotechnological production in heterologous hosts faces several constraints such as low efficiency of the corresponding enzymes or insufficient post-translational modifications^28,29^. Here, we present the extraction and identification of pentacyclic triterpenoids from *T. koksaghyz* NR and the functional characterization of pentacyclic triterpenoid-synthesizing genes in two heterologous hosts, namely *N. benthamiana* and *S. cerevisiae*. This knowledge is an essential prerequisite for establishing bioengineering approaches to produce pentacyclic triterpenoids and develop NR with more defined properties.

We performed a comprehensive analysis of components in acetone extract of *T. koksaghyz* NR. In contrast to previous analyses of acetone extracts from *T. koksaghyz* root material^30^, which supposedly contained lupeol and β-amyrin as prevailing triterpenes, we were able to achieve a far more detailed and differentiated elucidation of pentacyclic triterpenes in NR acetone extracts. Accordingly, we were able to show that at least 18 pentacyclic triterpenes and triterpenoids are present, with the most abundant pentacyclic triterpenes being taraxasterol (45%) and β-amyrin (13%). We also identified a so far unknown pentacyclic triterpene, called lup-19(21)-en-3-ol, that represents about 6% of all triterpene compounds. An additional novel finding is that about 14% of the triterpene extracts comprise ketone derivatives, including taraxasterone, β-amyrone and lup-19(21)-en-3-one. Furthermore, we were able to successfully apply a newly developed two-step HPLC method to separate the single triterpenes and triterpenoids from each other and purify the compounds with a high purity, ranging from 96% up to 100%.

Apart from the lupeol synthases ToLUP^31^ and TkLUP^15^, genes encoding triterpene-generating or -modifying enzymes from dandelion have not yet been functionally characterized. We were able to isolate six additional *OSC* cDNA sequences from dandelion latex and root tissue. In a cladogram, the corresponding cDNA-derived protein sequences of OSCs clustered widely among other OSC proteins of various functions (Fig. 2a). In conjunction with the diverse spatial expression patterns exhibited by *OSC* genes (Fig. 3a), this could indicate a sub-functionalization as observed for *A. thaliana OSC* genes *AtPEN1*, *AtPEN4* and *AtPEN5*, all synthesizing substantially different triterpenes^32–34^. Interestingly, the closely related *TkOSC1* and *TkOSC2* as well as *TkOSC5* were predominantly expressed in latex and, therefore, were the most promising enzymes for controlling the synthesis of pentacyclic triterpenes occurring in NR. While TkOSC2 and TkOSC5 did not show enzyme activity in the *N. benthamiana* expression system, TkOSC1 exhibited a mixed OSC function, as has also been described for AaOSC2 which is clustered closely to TkOSC1 and TkOSC2^12^. This illustrates that only slight sequence differences can massively influence enzyme functionality, as reviewed by Hoshino^35^, who described the β-amyrin synthase EtbAS derived from *Euphorbia tirucalli*. In that study, site-directed mutagenesis M729N of this monofunctional OSC led to formation of deviating end-products such as lupeol and germanicol^36^. The identical amino acid substitution appears in TkOSC1 and TkOSC2. In case of TkOSC5, missing enzyme activity might be due to a modified substrate binding motif (DTTAE instead of DCTAE) as shown in Supplementary Fig. S2. TkLUP as the predominant OSC in leaf tissue mainly produces lupeol, while TkOSC6 whose gene expression is highest in peduncle exhibited a high β-amyrin synthase activity, which is in accordance with TkLUP being closely related to AaLUP and TkOSC6 to AabAS, respectively (Fig. 2a).

For expression of *TkOSC1*, *TkOSC6* and *TkLUP* in *N. benthamiana*, we were able to increase the flux of precursors through the MVA pathway by overexpressing a truncated *Tkhmgrc1* comprising the catalytic domain only. This is in accordance with infiltration of a truncated oat *HMGR* which also led to increased triterpenoid levels in *N. benthamiana*^37,38^.

TkOSC1 generates at least seven different products that were also detected in the NR acetone extract, including the main components taraxasterol and β-amyrin as well as α-amyrin and the newly identified lup-19(21)-en-3-ol. This demonstrates the pivotal role of TkOSC1 for synthesis of the predominant pentacyclic triterpenes in *T. koksaghyz* latex.

To date, only six other OSCs from *A. thaliana* (AtLUP1, AtLUP2, AtBARS1 and AtPEN6)^37^, *Pisum sativum* (PsOSCPSM)^38^ and *Solanum lycopersicum* (SlTTS2)^39^ have been identified to generate taraxasterol, in most cases only in trace amounts, according to the newly established TriForC database^40^. Thus, employing TkOSC1 for efficient taraxasterol production could be advantageous, especially because this pentacyclic triterpene has shown anti-tumour and anti-carcinogenic activities^41–46^.

The triterpene-modifying enzyme CYP716A263 clusters among other CYP716A subfamily members (Fig. 2b) and shows an extraordinarily high expression in latex tissue of *T. koksaghyz* (Fig. 3b). Thus, we conclude that it exhibits a pivotal activity in this lipophilic tissue, comparable to CYP716A14v2 highly expressed in trichomes (Moses *et al*.^12^).

Hitherto, P450 enzymes have been capable of oxidizing C-3, C-6, C-12, C-16, C-22 and most commonly, C-28 positions of pentacyclic triterpenes^16,47–49^. Still, to our knowledge, a C-3 oxidation activity of CYP716 family members has only previously been observed for CYP716A14v2 from *A. annua* (Moses *et al*.^12^). As glucosyltransferases preferentially catalyse the addition of sugar moieties to C-3 and C-28 hydroxyl groups of pentacyclic triterpenes^50^, C-3-oxidizing reactions executed by CYP716A263 render these modified triterpenoids inaccessible and putatively sequester them to the lipophilic environment in latex. Consequently, the bioactive function of these ketone triterpenoids, putatively in defence against pathogens, will be addressed in future studies. Additionally, some pentacyclic triterpenes exist that do contain a C-3 carbonyl group: Shionone has been isolated from *Aster tataricus* and friedelin has been isolated from diverse species^51–53^. For those compounds, generation of the ketone derivatives is catalysed by the corresponding OSC in a single enzyme reaction, whereas the pentacyclic triterpenoids containing a C-3 carbonyl group isolated from *T. koksaghyz* NR in the current study are C_30_H_48_O isomers. Two successive enzymes, either TkLUP, TkOSC1 or TkOSC6 in combination with the oxidizing CYP716A263, synthesize them; this process coincides with that of amyrones detected in *A. annua* (Moses *et al*.^12^).

However, the coexpression of CYP716D60 with OSCs did not produce any additional pentacyclic triterpenoids in the heterologous *N. benthamiana* system. This might be attributed to low activity, unfavourable expression conditions or inadequate substrates for CYP716D60. Similarly, no activity was reported for CYP716D22 from *A. annua*. However, other CYP716D subfamily members such as CYP716D from *Stevia rebaudiana*^54^ have been shown to catalyse oxidation reactions of diterpenes, which could be evaluated in future studies.

Concerning CYP716A263, we were able to prove the rare C-3 oxidation of taraxasterol, lupeol and β-amyrin. To our knowledge, this is the first taraxasterol-oxidizing activity reported for a P450 enzyme. Due to limited P450 activity in the utilized heterologous systems, only minor amounts of the products could be obtained which might have impeded identification of further products. Consequently, oxidation of lup-19(21)-en-3-ol and α-amyrin by CYP716A263 seem possible under optimal conditions. Moreover, these products could be toxic at higher concentrations in yeasts or tobacco cells, as sequestration might not be as effective as in *T. koksaghyz* latex. Nonetheless, various approaches, such as modifying the *CYP*/*AtR2* expression ratio as previously proposed^55^, increasing CYP716A263 efficiency by employing as-yet unknown native CPR from *T. koksaghyz*, or modifying the N-terminus, could help elucidate whether CYP716A263 is also capable of oxidizing lup-19(21)-en-3-ol, α-amyrin and other pentacyclic triterpenes present in *T. koksaghyz* latex. To our knowledge, this is the first identification of a promiscuous P450 enzyme that oxidizes pentacyclic triterpenes at the C-3 position in latex tissue.

Moreover, this study offers new insight into dandelion resin composition possibly affecting NR properties, fostering industrially relevant approaches to separate dandelion NR.

## Methods

### Plant material and cultivation conditions

*T. koksaghyz* wild-type plants were cultivated at 18 °C and 20 klux with a 16-h photoperiod in controlled growth chambers or in a greenhouse. Plants were cultivated in a pre-fertilized 1:1 mixture of standard soil (ED73 Einheitserde, Fröndenberg, Germany) and garden mould (Botanical Garden Münster, Germany). They were fed every 4 weeks with a commercial fertilizer according to the manufacturer’s recommendations (Hakaphos Plus, Compo GmbH, Münster, Germany). Seeds of *Nicotiana benthamiana* were obtained from the Sainsbury Laboratory (John Innes Centre, Norwich, United Kingdom) and cultivated as stated above.

### Total RNA extraction and cDNA synthesis

Total RNA was extracted from *T. koksaghyz* latex, root, leaf, peduncle and flower tissues using the innuPREP RNA Mini Kit (Analytik Jena, Jena, Germany) according to the manufacturer’s instructions. Full-length cDNA was synthesized from 500 ng total RNA using PrimeScript RT Master Mix (TaKaRa, Clontech, Saint-Germain-en-Laye, France) according to the manufacturer’s instructions.

### *In silico* and phylogenetic analysis

Amino acid sequences were obtained from GenBank (https://www.ncbi.nlm.nih.gov/genbank/) and are listed in Supplementary Table S3. Conserved protein domains were determined using PROSITE (http://prosite.expasy.org/), and transmembrane domains were predicted using TMHMM software (http://www.cbs.dtu.dk/services/TMHMM-2.0/). Multiple alignments of protein sequences were generated using the Clustal MUSCLE algorithm^56^ (http://www.ebi.ac.uk/Tools/msa/muscle/), while cladograms were created using MEGA6^57^ (http://www.megasoftware.net/).

### Cloning procedures

For the cloning of *N. benthamiana* infiltration constructs, *TkLUP*, *TkOSC1*, *TkOSC2*, *TkOSC5* and *TkOSC6* full-length cDNAs were amplified with the primers TkLUP-fwd-SalI/TkLUP-rev-XhoI, TkOSC1-fwd-NcoI/TkOSC1-rev-XhoI, TkOSC2-fwd-NcoI/TkOSC2-rev-XhoI, TkOSC5-fwd-SalI/TkOSC5-rev-XhoI, TkOSC6-fwd-SalI/TkOSC6-rev-XhoI, restricted with the corresponding enzymes and inserted into the SalI/XhoI and NcoI/XhoI sites of the Gateway (GW) vectors pENTR3c and pENTR4, respectively (Thermo Fisher Scientific, Waltham, MA, USA), resulting in the vectors pENTR3c-TkLUP, pENTR4-TkOSC1, pENTR4-TkOSC2, pENTR3c-TkOSC5 and pENTR3c-TkOSC6. The *TkLUP*, *TkOSC1*, *TkOSC2*, *TkOSC5* and *TkOSC6* sequences were subsequently introduced into the GW-compatible vectors pBatTL-ccdB^58^ by LR recombination (Thermo Fisher Scientific, Waltham, MA, USA). The vectors pBatTL-TkLUP, pBatTL-TkOSC1, pBatTL-TkOSC2, pBatTL-TkOSC5 and pBatTL-TkOSC6 were used for *Agrobacterium*-mediated infiltration of *N. benthamiana* leaves.

The *CYP716A263* and *CYP716D60* full-length cDNA was amplified using the primers CYP716A263-fwd-NcoI/CYP716A263-rev-XhoI and CYP716D60-fwd-BamHI/CYP716D60-rev-XhoI, respectively. The resulting PCR products were digested with appropriate enzymes and inserted into the NcoI/XhoI and BamHI/XhoI sites of the Gateway (GW) vector pENTR4 (Thermo Fisher Scientific, Waltham, MA, USA), yielding the vectors pENTR4-CYP716A263 and pENTR4-CYP716D60. For heterologous expression studies in *N. benthamiana*, the *CYP716A263* and *CYP716D60* sequences were introduced into the GW-compatible vectors pBatTL-ccdB by LR recombination (Thermo Fisher Scientific, Waltham, MA, USA) to generate the constructs pBatTL-CYP716A263 and pBatTL-CYP716D60 suitable for *Agrobacterium*-mediated infiltration.

Similarly, amplifying *Tkhmgrc1* with the oligonucleotides Tkhmgrc1-fwd-PciI/Tkhmgrc1-rev-XhoI and inserting the restricted PCR product into the NcoI/XhoI sites of pENTR4 gave the pENTR4-Tkhmgrc1. Subsequently, LR recombination into the GW-compatible vector pBatTL-ccdB yielded pBatTL-Tkhmgrc1.

For heterologous expression constructs suitable for yeast, the *TkLUP*-coding sequence from pENTR3c-TkLUP was introduced into pAG424Gal1-ccdB^59^ via LR recombination (Thermo Fisher Scientific, Waltham, MA, USA), yielding pAG424Gal1-TkLUP^15^. For *TkOSC1* expression in yeast, a fusion construct of the TkLUP N-terminus and the TkOSC1 C-terminus had to be cloned. Therefore, TkLUP N-terminus was amplified with the primers TkLUP-fwd-Sc-SalI and TkLUP-rev-Sc-Nterm, comprising a sequence of 63 bp. The TkOSC1 C-terminus was amplified using the primers TkOSC1-fwd-Sc-Cterm, containing an overhang sequence to the TkLUP N-terminus sequence, and TkOSC1-rev-XhoI. The resulting fragments were combined in an overlap extension PCR conducted with the primers TkLUP-fwd-Sc-SalI and TkOSC1-rev-XhoI, restricted with SalI/XhoI and inserted into the SalI/XhoI site of the Gateway (GW) vector pENTR3c, yielding pENTR3c-TkLUPN-TkOSC1C. The TkLUPN-TkOSC1C coding sequence was introduced into pAG424Gal1-ccdB^59^ via LR recombination (Thermo Fisher Scientific, Waltham, MA, USA), yielding pAG424GAL1-TkLUPN-TkOSC1C. Expression constructs suitable for yeast were generated for the *TkOSC6*-coding sequence. Here, the oligonucleotides TkOSC6-fwd-Sc-KpnI/TkOSC6-rev-XhoI were used to amplify the full-length sequence, which was restricted with the corresponding enzymes and inserted into the KpnI/XhoI site of the Gateway (GW) vector pENTR3c (Thermo Fisher Scientific, Waltham, MA, USA), resulting in the vector pENTR3c-TkOSC6-Sc. Subsequently, the TkOSC6-Sc sequence was introduced into pAG424Gal1-ccdB^59^ via LR recombination (Thermo Fisher Scientific, Waltham, MA, USA), yielding pAG424Gal1-TkOSC6. Sequencing validated all constructs. Subsequently, yeast strain CEN.PK2-1C (MATa; his3D1; leu2-3_112; ura3-52; trp1-289; MAL2-8c; SUC2)^60^ harboring several other modifications (rox1::P_GAL1_-tHMGR P_GAL10_-ERG13; P_ERG7_Δ::P_CTR3_)^15^ was transformed with pAG424Gal1-TkLUPN-TkOSC1C and pAG424Gal1-TkOSC6. The resulting yeast strains CEN.PK2-1C (rox1::P_GAL1_-tHMGR P_GAL10_-ERG13; P_ERG7_Δ::P_CTR3_; P_GAL1_-TkLUPN-TkOSC1C) and CEN.PK2-1C (rox1::P_GAL1_-tHMGR P_GAL10_-ERG13; P_ERG7_Δ::P_CTR3_; P_GAL1_-TkOSC6) were used for expression analyses and subsequent triterpene extractions.

For expression of *CYP716A263* in yeast, the vector 1B3-pESC-Ura had to be generated. The pESC-URA cassette (Agilent Technologies, Santa Clara, USA) consisting of ADH-T, MCS and the bidirectional Gal promoter was amplified with the primers pESC-URA-fwd/pESC-URA-rev, restricted with XhoI and inserted into the XhoI/EcoICRI site of GW-compatible vector 423GPD-ccdB (1B3; Addgene, Cambridge, MA, USA), thus yielding 1B3-pESC-URA. *CYP716A263* cDNA sequence was amplified using the primers CYP716A263-fwd-BamHI and CYP716A263-rev-XmaI, restricted with the corresponding enzymes and inserted into the BamHI/XmaI site of 1B3-pESC-URA, yielding 1B3-pESC-URA-CYP716A263. Subsequently, *AtR2* from *A. thaliana* was amplified using the primers AtR2-fwd-NotI and AtR2-rev-SpeI, digested with NotI/SpeI and inserted into the NotI/SpeI cassette of 1B3-pESC-URA-CYP716A263, thus generating the yeast vector 1B3-pESC-URA-CYP716A263-AtR2 validated by sequencing and used for transformation into CEN.PK2-1C (rox1::P_GAL1_-tHMGR P_GAL10_-ERG13; P_ERG7_Δ::P_CTR3_; P_GAL1_-TkLUPN-TkOSC1C). The resulting yeast strain CEN.PK2-1C (rox1::P_GAL1_-tHMGR P_GAL10_-ERG13; P_ERG7_Δ::P_CTR3_; P_GAL1_-TkLUPN-TkOSC1C; P_GAL1_-CYP716A263; P_GAL10_-AtR2) was cultivated and used for triterpene extractions. All nucleotide sequences used for cloning are shown in Supplementary Table S4.

### Quantitative RT-PCR (qRT-PCR)

Quantitative RT-PCR analysis was carried out as previously described^7^. *T. koksaghyz* wild-type plants were grown for 12 weeks for spatial expression analyses. RNA was extracted from nine individual plants, before the cDNA of three plants each was pooled. All oligonucleotide sequences for the expression analysis are shown in Supplementary Table S4. Primer efficiencies and amplification factors are shown in Supplementary Table S5.

### Heterologous expression in *N. benthamiana*

Infiltration of *N. benthamiana* was carried out as previously described^25^ with a slight modification: FAD (Sigma-Aldrich, Taufkirchen, Germany) was provided as a cofactor in the infiltration (end-concentration of 0.05 mM). pBatTL constructs were infiltrated in the following combinations: pBatTL-Tkhmgrc1, pBatTL-Tkhmgrc1 + pBatTL-TkLUP; pBatTL-Tkhmgrc1 + pBatTL-TkLUP + pBatTL-CYP716A263; pBatTL-Tkhmgrc1 + pBatTL-TkLUP + pBatTL-CYP716D60; pBatTL-Tkhmgrc1 + pBatTL-TkOSC1; pBatTL-Tkhmgrc1 + pBatTL-TkOSC1 + pBatTL-CYP716A263; pBatTL-Tkhmgrc1 + pBatTL-TkOSC1 + pBatTL-CYP716D60; pBatTL-Tkhmgrc1 + pBatTL-TkOSC2; pBatTL-Tkhmgrc1 + pBatTL-TkOSC2 + pBatTL-CYP716A263; pBatTL-Tkhmgrc1 + pBatTL-TkOSC2 + pBatTL-CYP716D60; pBatTL-Tkhmgrc1 + pBatTL-TkOSC5, pBatTL-Tkhmgrc1 + pBatTL-TkOSC5 + CYP716A263, pBatTL-Tkhmgrc1 + pBatTL-TkOSC5 CYP716D60; pBatTL-Tkhmgrc1 + pBatTL-TkOSC6, pBatTL-Tkhmgrc1 + pBatTL-TkOSC6 + CYP716A263, pBatTL-Tkhmgrc1 + pBatTL-TkOSC6 CYP716D60. After 7 days of incubation the infiltrated leaves were freeze-dried, ground and subjected to triterpene extraction as previously described^25^.

### Heterologous expression in *S. cerevisiae*

Yeast transformation was carried out as previously described^61^. For expression of galactose-inducible genes a single colony was picked, inoculated in 5 mL SD medium and cultivated overnight at 30 °C and 130 rpm. From this culture, 100 mL of fresh SD medium (containing 150 µM CuSO_4_ for repression of the *erg7* expression) where inoculated to a final cell density of 10^5^ cells mL^−1^ and grown at 30 °C and 130 rpm in a 500 mL Erlenmeyer flask. When the culture reached a cell density of 2 × 10^6^ cells mL^−1^ the medium was changed to SD medium containing galactose instead of glucose to induce gene expression. The yeast cells were grown until a cell density of 4 × 10^6^ cells mL^−1^ was reached and harvested by centrifugation (10 min., 1000 × *g*). After harvesting, 100 µL of internal standard cholesterol (1 mg mL^−1^ in acetone) were added. Cells were lyophilized for four days until triterpene extraction from pelleted cells was performed according to extraction from leaf material as previously described^25^.

### Triterpene purification by HPLC

We utilized a bead milling process to purify raw rubber from *T. koksaghyz* roots^62^ and extracted therein-comprised lipids by acetone for 7 days at room temperature. Semi-preparative HPLC of the lipid extract was carried out using a Shimadzu LC20A HPLC system (Shimadzu, Duisburg, Germany) coupled to a UV detector (SPD-M20A) and a fraction collector (FRC-10A). The triterpenes were separated using an Ultra C18 column (250 × 21.2 mm, particle size: 5 µm, Restek GmbH, Bad Homburg, Germany) and methanol as solvent with a flow rate of 10 ml min^−1^. The column oven temperature was set to 40 °C. Detection was carried out at 205 nm and the triterpene fractions were collected, dried using Rocket evaporator system (Thermo Fisher Scientific), dissolved in acetone and analysed by GC-MS. In a second purification step, an Ultra Biphenyl column was used as a stationary phase (250 × 21.2 mm, particle size: 5 µm, Restek GmbH, Bad Homburg, Germany). The column oven temperature was set to 40 °C and the triterpenes were separated with a gradient of methanol (A) and water (B) at a flow rate of 8 ml min^−1^ using the following elution profile: 0–25 min, isocratic 90% A; 25–71 min, linear from 90% to 100% A; 71–75 min, isocratic 100% A; followed by column re-equilibration: 75–76 min, linear from 100% to 90% A; 76–85 min, isocratic 90% A. Triterpenes were identified by GC-MS as previously described^7^ using standard compounds (β-amyrin, α-amyrin, lupeol and lupenone were purchased from Extrasynthese, Genay, France; Taraxerol and β-amyrone from Sigma-Aldrich, Taufkirchen, Germany). For quantification, the fractions of one HPLC-run (C18-column) were collected, dried, dissolved in 1 ml of acetone and analysed by GC-MS. Peak areas of total ion counts (TICs) were used for calculating the percentage amount of the single triterpenes.

### NMR spectroscopy

The triterpenes were dissolved in 140 μL of CDCl_3_, transferred to Bruker Match tubes and subjected to one- and two-dimensional NMR analysis. ^13^C NMR spectra were measured with a Bruker Avance-III 500 MHz spectrometer equipped with a cryo probe (5 mm CPQNP, 1H/13C/31P/19F/29Si; Z-gradient). ^1^H NMR spectra were registered with an Avance-I 500 MHz system and an inverse probe head (5 mm SEI, 1H/13C; Z-gradient). The temperature was 300 K. Data processing and analysis was done with TOPSPIN 3.0 or MestreNova. The one-dimensional ^1^H and ^13^C NMR including DEPT90 and DEPT135 spectrum as well as COSY, TOCSY, HSQC, HMBC and NOESY spectra were measured with standard Bruker parameter sets. Due to low amounts of isolated lup-19(21)-en-3-ol and its ketone, only a limited set of high intensity correlation signals could be observed in COSY, HSQC and HMBC experiments (Supplementary Table S1). Lup-19(21)-en-3-ol and its ketone could be identified based on published NMR data of the acetate derivative^63^.

### Accession numbers

TkOSC1 (MG646376), TkOSC2 (MG646377), TkOSC3 (MG646378), TkOSC4 (MG646379), TkOSC5 (MG646380), TkOSC6 (MG646381), CYP716A263 (MG646382), CYP716D60 (MG646383).

## Supplementary information

Supplementary Information

## Data Availability

All data generated during and analysed during this study are included in this published article (and its Supplementary Information Files).
