# Supplementary material for: The enzymes OSC1 and CYP716A263 produce a high variety of triterpenoids in the latex of Taraxacum koksaghyz
Source: Sci Rep. 2019 Apr 11;9:5942. doi: 10.1038/s41598-019-42381-w (PMC6459903; doi:10.1038/s41598-019-42381-w)
Supplement: Supplementary file 1 — Supplementary Information [file 41598_2019_42381_MOESM1_ESM.docx]

## *Scientific Reports* Supplementary Information

Article title: The enzymes OSC1 and CYP716A263 produce a high variety of triterpenoids in the latex of *Taraxacum koksaghyz*

Authors: Katharina M. Pütter, Nicole van Deenen, Boje Müller, Lea Fuchs, Kirsten Vorwerk, Kristina Unland, Jan Niklas Bröker, Emely Scherer, Claudia Huber, Wolfgang Eisenreich, Dirk Prüfer and Christian Schulze Gronover

The following Supplementary Information is available for this article:

**Supplementary Figure S1** GC-MS data for HPLC fractions separated by Ultra C18 column.

**Supplementary Figure S2** *In silico* analysis of TkLUP, TkOSC1-6, CYP716A263 and CYP716D60 from *T. koksaghyz*.

**Supplementary Figure S3** Heterologous expression of *T. koksaghyz* *OSC* and *CYP716D60* in *N. benthamiana*.

**Supplementary Figure S4** Heterologous expression of *T. koksaghyz* *TkOSC6* and *CYP716A263* in *N. benthamiana* and *S. cerevisiae*.

**Supplementary Figure S5** Heterologous expression of *T. koksaghyz* *TkOSC1* in *S. cerevisiae*.

**Supplementary Table S1** NMR data of lup-19(21)-en-3-ol and lup-19(21)-en-3-one.

**Supplementary Table S2** Properties of identified *OSC* and *P450* genes from *T. koksaghyz*.

**Supplementary Table S3** Protein sequence data and corresponding accession numbers obtained from GenBank.

**Supplementary Table S4** Sequences of oligonucleotides used for cloning and qRT-PCR.

**Supplementary Table S5** Primer efficiency and amplification factors for cDNA obtained from *T. koksaghyz* mRNA.

**Supplementary Figure S1.** **GC-MS data for HPLC fractions separated by Ultra C18 column**. GC chromatograms of fractions 1-7 (F1-F7) and the mass spectra of the main peaks are shown. All triterpenes that could be clearly identified by the NIST library, corresponding standard compounds or by NMR analysis are named in the mass spectra. Taraxasterol and taraxasterone were identified by using GC-MS and NMR data obtained from PubChem compound database (https://pubchem.ncbi.nlm.nih.gov).


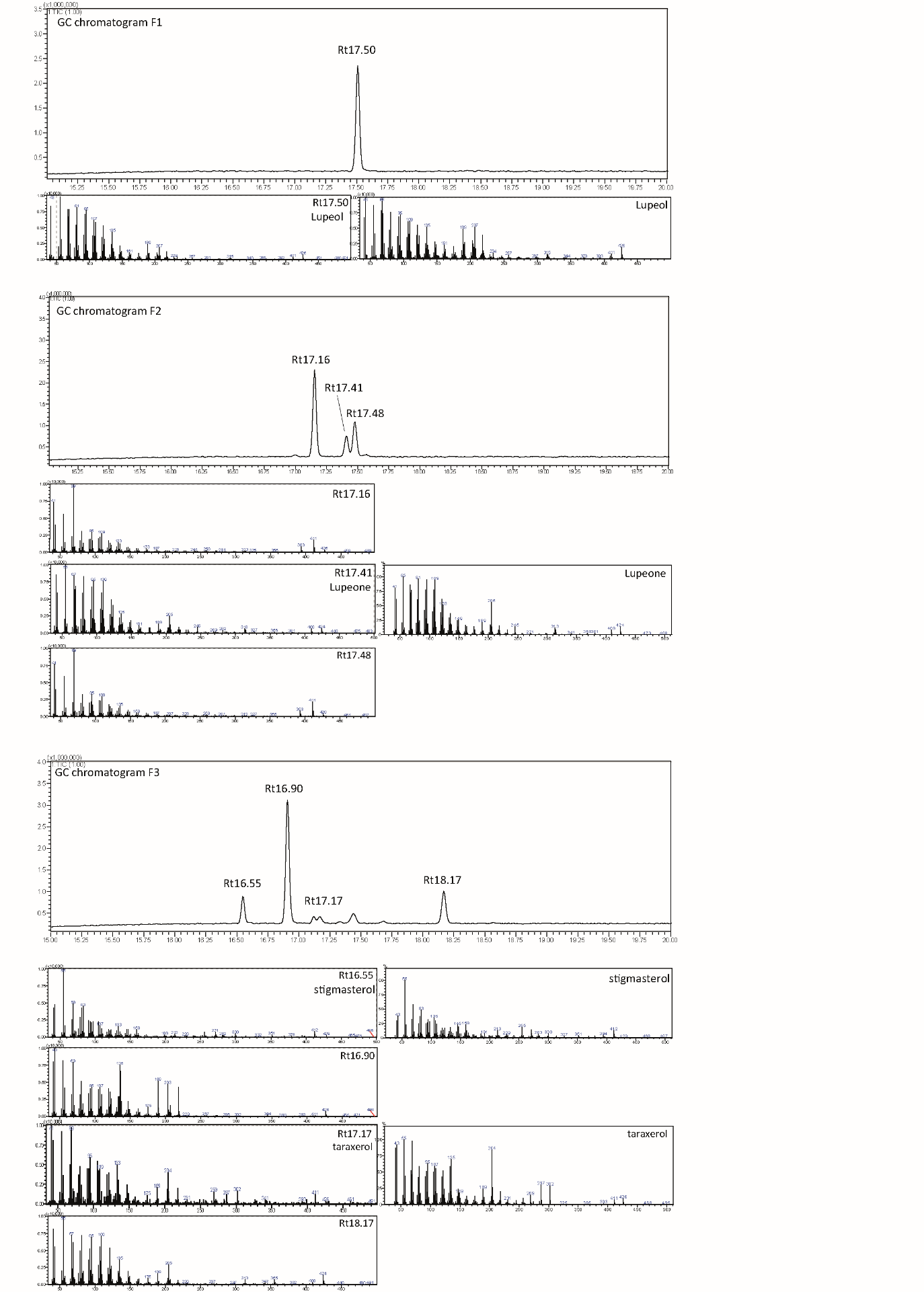


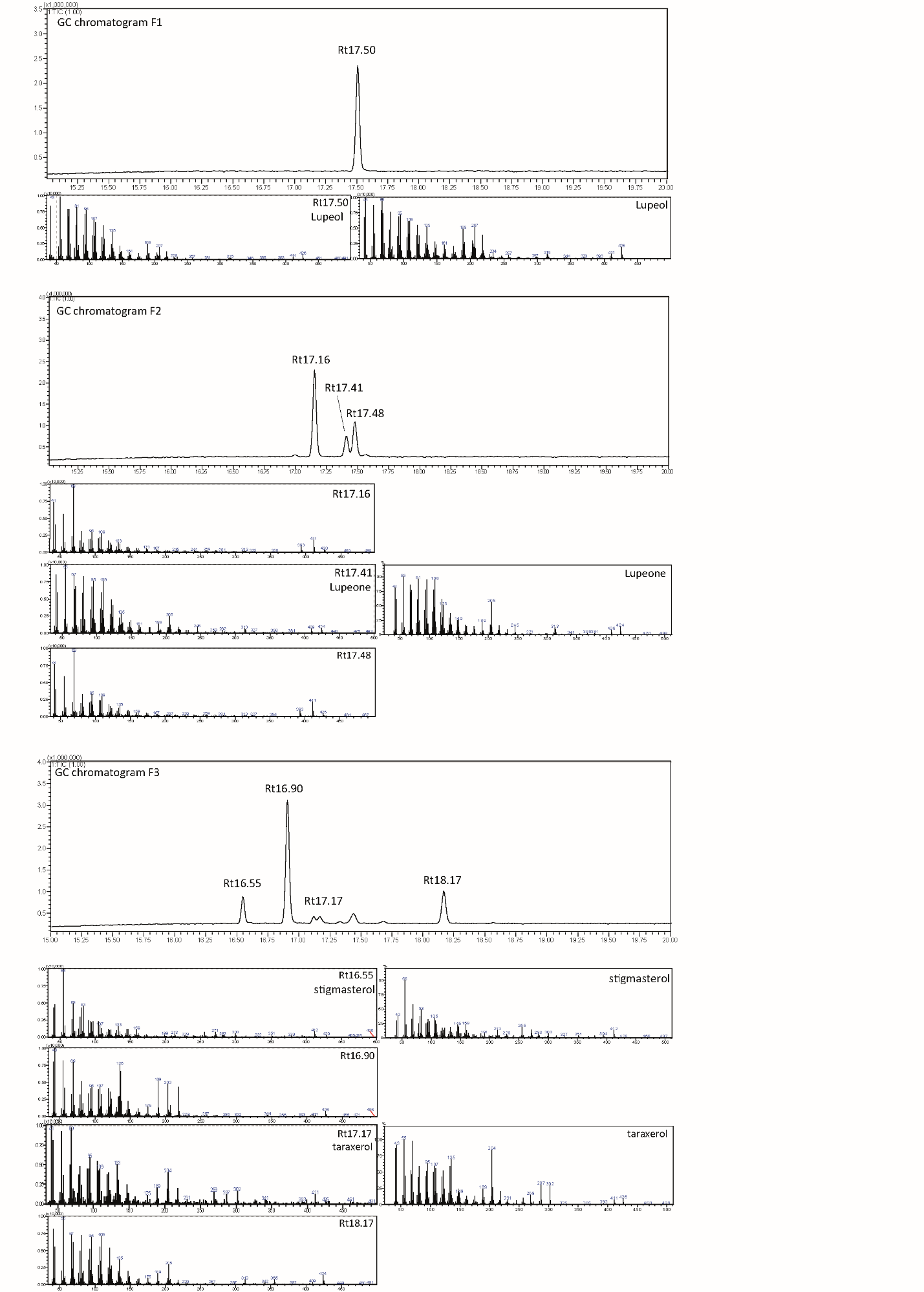


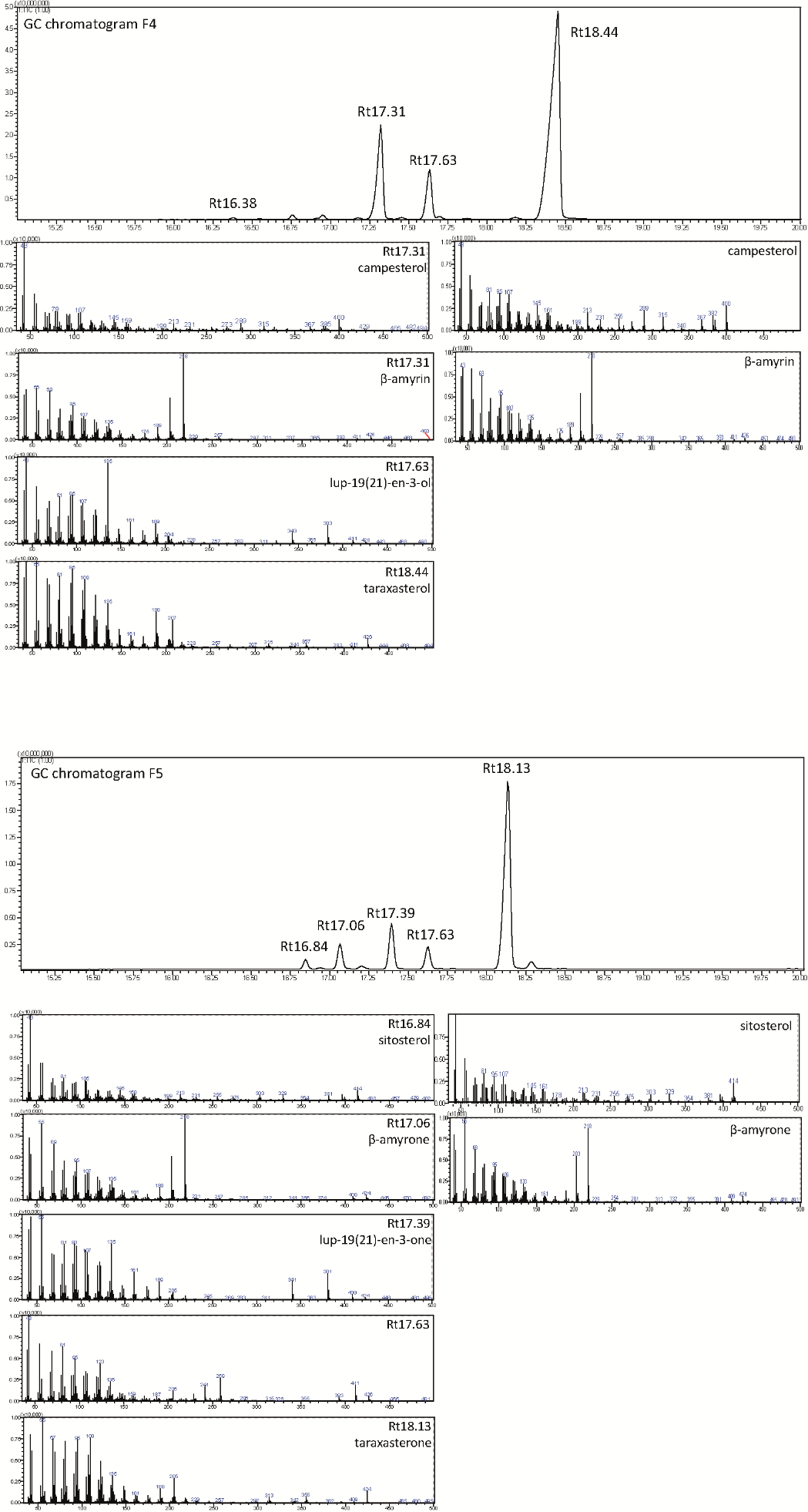


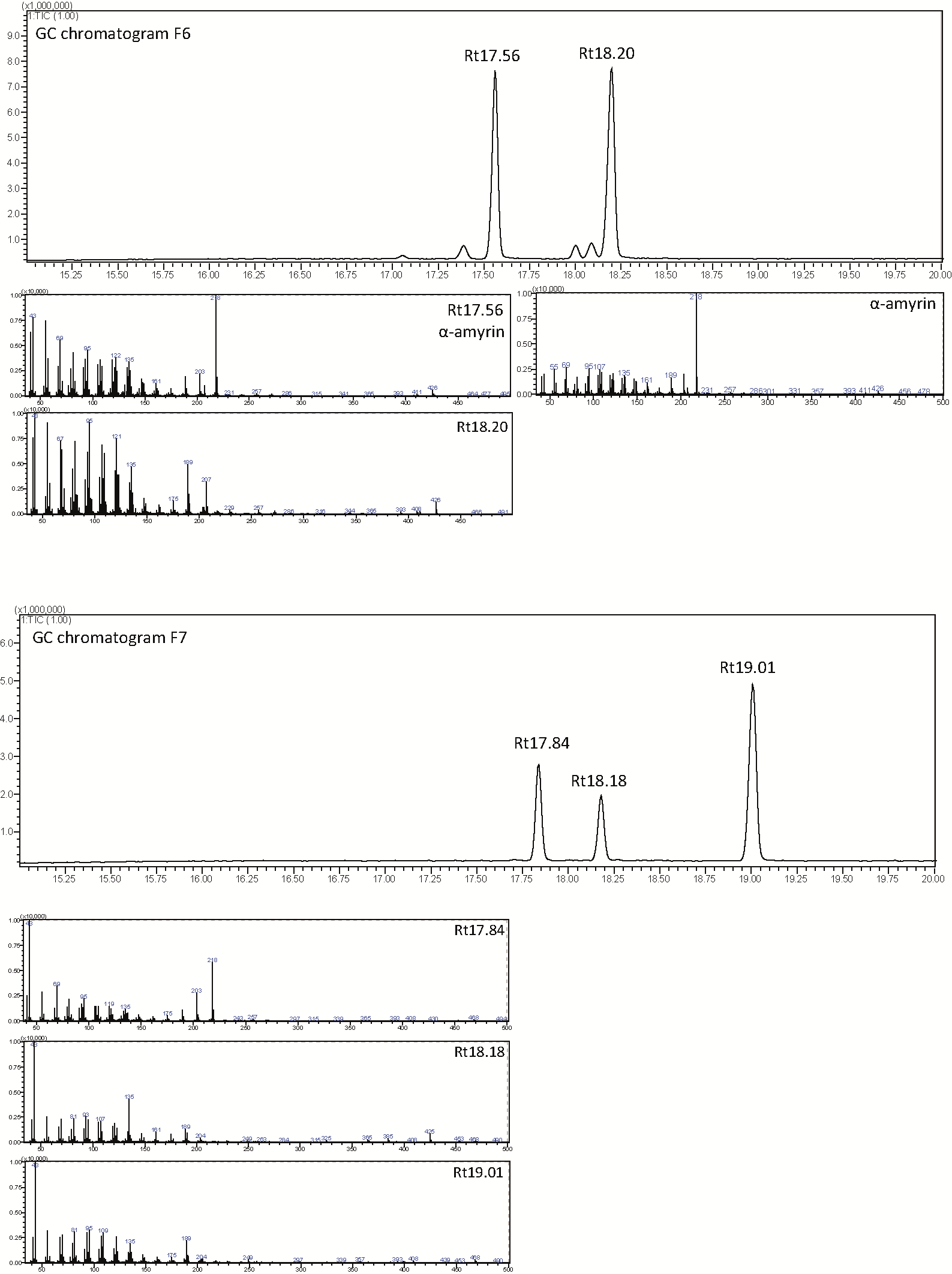


**Supplementary Figure S2.** ***In silico* analysis of TkLUP, TkOSC1-6, CYP716A263 and CYP716D60 from *T. koksaghyz*.** Alignments were created with Clustal MUSCLE^58^ (http://www.ebi.ac.uk/Tools/msa /muscle/). Dashes represent gaps in aligned sequences, predicted transmembrane domains are underlined and conserved amino acids are shown in boldface. Transmembrane domains were predicted using TMHMM software (http://www.cbs.dtu.dk/services/TMHMM-2.0/). Protein sequences were obtained from GenBank (https://www.ncbi.nlm.nih.gov/genbank/). (a) TkOSC1-6 and TkLUP amino acid alignment with OSC and LUP sequences from *A. annua* (AaLUP, AJE29379.1; AaOSC2, AHF22084.1), *A. thaliana* (AtLUP1, NP_178018.1; AtLUP2, NP_178017.2), *O. europaea* (OeOSC, BAF63702.1), *S. lycopersicum* (SlOSC, NP_001234597.1) and *T. officinale* (ToLUP, BAA86933.1). Conserved sites include a domain for product determination (M(W/Y)CY(C/S)R motif, blue), a region involved in substrate binding and cyclization (DCTAE motif, green) and repeated QW motifs (yellow) involved in stabilization of carbocationic intermediates which are localized near the 5’ and 3’ end of the proteins. (b) CYP716A263 and CYP716D60 amino acid alignment with P450 sequences from *A. annua* (CYP716A14v2, AHF22083.1; CYP716D22, AHF22082.1), *A. thaliana* (CYP716A1, NP_193268.3), *B. platyphylla* (CYP716A180, AHL46848.1), *M. truncatula* (CYP716A12, CBN88268.1), *V. vinifera* (CYP716A15, NP_001268115.1). Conserved sites include the hydrophobic N-terminal transmembrane domain (underlined), a proline-rich region (yellow, consensus sequence PPGxxGxP), the I-helix involved in oxygen binding (blue, consensus sequence A/GGxD/ET), the E-R-R triad (consisting of the ExxR motif (K-helix, green) and the PERF motif (pink), respective residues shown in boldface) involved in catalysis and stabilization of the core structure and the heme-binding motif (grey, consensus sequence FxxGxxxCxG, PROSITE accession number PS00086) near the C-terminus.

**(a)**

TkOSC1 MWKLRIGEKNGKFNIGDGNGDD-YLHSTNNFVGRQTWEFDPD-AGTQKERDEIERLREQF 58

TkOSC2 MWKLRIGEKNGKFNIGDGNGDE-YLYSTNNFVGRQTWEFDPD-AGTQEERDEVERLREQF 58

TkOSC3 MWELKIAE---------G--DGPYLYSTNNFVGRQFWEFNPD-AGTPEEKEEIEKVRQKF 48

AaOSC2 MWKLKVAE---------G--NDPYLFSTNNFVGRQIWEFDPS-AGSPVERQEVEDARQQF 48

OeOSC MWKLKIAE---------G--HGPYLYSTNNFAGRQIWEYDPN-GGTPEEREAYDKAREEF 48

AaLUP MWKLKIAE---------GAGDK-WLSTTNNHIGRQHWEFDPD-AGTEEERAEIERIRLNF 49

TkLUP MWKLKIAE---------G-SDDEWLTTTNNHVGRQHWQFDPD-AGTEEERAQIEKIRLNF 49

ToLUP MWKLKIAE---------G-GDDEWLTTTNNHVGRQHWQFDPD-AGTEEERAEIEKIRLNF 49

TkOSC4 MWRLRIGQ---------GNKDDPYLFSTNNFVGRQIWEYDENYKATPEELEEVKQARSSF 51

AtLUP1 MWKLKIGK---------GNGEDPHLFSSNNFVGRQTWKFDHK-AGSPEERAAVEEARRGF 50

AtLUP2 MWKLKIGE---------GNGEDPYLFSSNNFVGRQTWEFDPK-AGTPEERAAVEDARRNY 50

SlOSC MWKLKIAK---------G-QDDRYLYSTNNYIGRQIWEFDPN-AGTIEEQAKIEEARQHY 49

TkOSC5 MWKLKIAE---------G-GNDPYLHSTNNFIGRQIWEFDSN-HGTPEDRAEAEHARINF 49

TkOSC6 MWKLKIAE---------G-GNDPYLHSTNNFIGRQIWEFDPN-HGTTEDRAEVEQARLDF 49

** *.:.: * * ::**. *** *::: . .: : . * :

TkOSC1 LINKKKLDIRCCADLLMRNQLIKESGIDLISVPPVRLGDDEDVNYEAVTTAVRKAVRLNR 118

TkOSC2 LINKKKLDISCCADLLMRNQLIKESGIDLISEAPVKLGHDEDVNYKAVTTAVRKAVRLNR 118

TkOSC3 KDNRKKGGFHACGDLLMRMQLMKENAIDLTSILPVRISEGEQVNYEATTIAVRKAVRLHR 108

AaOSC2 KNNRRE-GVHPCGDLLMRIQLIKENGIDVMSIPPVRLGENEDVNYDAVTTTVKKALRLNR 107

OeOSC QRNRKLKGVHPCGDLFMRIQLIKESGIDLMSIPPVRLGEKEEVTYETATTAVKKALLLNR 108

AaLUP KVNRFH--VKQSADLLMRMQLRKENPRAKI-PEAIKLKETEEVTNEAVTTTLRRAISFFS 106

TkLUP KLNRFQ--FKQSADLLMRTQLRKENPINKI-PDAIKLNETEEVTNDAVSTTLKRAISFYS 106

ToLUP KLNRFQ--FKQSADLLMRTQLRKENPINKI-PDAIKLNETEEVTNDAVTTTLKRAISFYS 106

TkOSC4 WNNRHK--QRPCDDRLWRFQFLREKNFKQT-IPREIIVDESEIKYETVDNALKRAVRYWT 108

AtLUP1 LDNRFR--VKGCSDLLWRMQFLREKKFEQG-IPQLKATNIEEITYETTTNALRRGVRYFT 107

AtLUP2 LDNRPR--VKGCSDLLWRMQFLKEAKFEQV-IPPVKIDDGEGITYKNATDALRRAVSFYS 107

SlOSC WNNRYK--VKPNSDLLWRMQFLREKNFKQR-IRAVKVEEGEEISHEIATVALHRAVHFFS 106

TkOSC5 WNLRHR--VKPSSDVLWRMQFLKEKQFKQT-IAQVKIEEFEEISYEKATVTLKRCVNLFA 106

TkOSC6 WNHRHQ--VKPSSDVLWRMQFLKEKEFKQT-IAQVKIEDSEEISYEKTTTTLRRCVSFFA 106

. * : * *: .* . :. . . ::.. :

TkOSC1 AI**Q**AWD**G**H**W**PAENAGPLFFTPPLIIALYISGTLDTILTQEHKREMIRYMYIH**Q**NED**G**G**W**G 178

TkOSC2 AI**Q**AWD**G**H**W**PAENAGPLFFTPPLIIVLYISGTLDNVLTQDHKKEMIRYMYIH**Q**NED**G**G**W**G 178

TkOSC3 GI**Q**AKD**G**H**W**PAENAGPLFFTPPLVIALYISGTINTVLSEEHKKEMIRYFYNH**Q**NED**G**G**W**G 168

AaOSC2 AI**Q**AKD**G**H**W**PAENAGPMFFTPPLLIAMYISGTINTHLTKEHRTEMIRYIYNH**Q**NED**G**G**W**G 167

OeOSC AV**Q**ASD**G**H**W**PAENAGPMFFTPPLIIVLYISGAINTILTSEHRKEMVRYIYNH**Q**NDD**G**G**W**G 168

AaLUP TI**Q**AHD**G**H**W**PAESAGPLFFLPPMVIALYMTGAMNNILTPAHQLEIKRYIYNH**Q**NED**G**G**W**G 166

TkLUP TI**Q**AHD**G**H**W**PAESAGPLFFLPPLVIALYVTGAMNDILTPAHQLEIKRYIYNH**Q**NED**G**G**W**G 166

ToLUP TI**Q**AHD**G**H**W**PAESAGPLFFLPPLVIALYVTGAMNDILTPAHQLEIKRYIYNH**Q**NED**G**G**W**G 166

TkOSC4 AL**Q**ASD**G**H**W**PSANNGCHYFTPPIVMCLYITGHLDTFFSTQDKEEMLRYIYCH**Q**NED**G**G**W**G 168

AtLUP1 AL**Q**ASD**G**H**W**PGEITGPLFFLPPLIFCLYITGHLEEVFDAEHRKEMLRHIYCH**Q**NED**G**G**W**G 167

AtLUP2 AL**Q**SSD**G**H**W**PAEITGTLFFLPPLVFCFYITGHLEKIFDAEHRKEMLRHIYCH**Q**NED**G**G**W**G 167

SlOSC AL**Q**ATD**G**H**W**PAESAGPLFFLPPLVMCMYITGHLNTVFPAEHRKEILRYIYCH**Q**NED**G**G**W**G 166

TkOSC5 AL**Q**ADD**G**H**W**PAENAGPLYFMQPLVICLYITGHLDIVFPEEHRKEILHYMYCH**Q**NED**G**G**W**G 166

TkOSC6 AL**Q**ASD**G**H**W**PAENAGPLYFMQPLVICLYITGHLNIVFPEEYRKEILRYLYCH**Q**NED**G**G**W**G 166

:*: *****. * :* *::: :*::* :: : . *: .::* ***:*****

TkOSC1 FYISGRSTMIGTALNYVGLRLLGEDDND-----AIAKGRKWILDHGGATSIPSWGKVYLS 233

TkOSC2 FYISGRSTMIGTALNYVGLRLLGEDDND-----AIAKGRKWILDHGGATSIPSWGKVYLS 233

TkOSC3 FYIEGHSTMIGSVLSYVALRLLGEGEDDG-DG-AIARARKWILDHGGAASIPSWGKVYLS 226

AaOSC2 FYIEGHSTMIGSALSYVALRLLGEGPDDG-NG-AVDRARKWILDHGGAASIPSWGKTYLS 225

OeOSC FYIEGHSTMIGSALSYIALRLLGEGPDDG-NG-SIARARKWILDHGGATGIPSWGKTYLS 226

AaLUP FHIEGHSTMFGSVYSYITLRLLGEEADSCAEDMAVVKGRKWILDHGGAVGTPSWGKFWLT 226

TkLUP LHIEGHSTIFGSVLSYITLRLLGEEADSVAEDMALVKGRKWILDHGGAVGIPSWGKFWLT 226

ToLUP LHIEGHSTIFGSVLSYITLRLLGEEADSVAEDMALVKGRKWILDHGGAVGIPSWGKFWLT 226

TkOSC4 FHLEGHSIMFCTAVNYICMRMLGEGPDGGLDG-ACSRARKWILDHGSVTAIPSWGKTWLS 227

AtLUP1 LHIESKSVMFCTVLNYICLRMLGENPE---QD-ACKRARQWILDRGGVIFIPSWGKFWLS 223

AtLUP2 LHIEGKSVMFCTVLNYICLRMLGEGPNGGRNN-ACKRARQWILDHGGVTYIPSWGKIWLS 226

SlOSC LHIEGHSTMFCTAMSYICMRILGEGPEGGVNN-ACARARKWILDHGSVIAIPSWGKTWLS 225

TkOSC5 FHIEGHSSMFCTTLSYICMRLLGEGVDGGLNG-ACTKARKWILDHGSVTAIASWGKTWLS 225

TkOSC6 FHIEGHSTMFGTTLSYICMRLLGEGPDGGLNG-ACTKARKWILDHGSVTAIPSWGKTWLS 225

:::...* :: :. .*: :*:*** : : ..*:****.*.. .**** :*:

TkOSC1 VLGVYEWAGCNPLPPEFWLFPSFLPYHPAK**MWCY**C**R**TTYMPMSYLYGTGFQGPITDLVKS 293

TkOSC2 VLGVYEWAGCNPLPPEFWLFPSFFPYHPAK**MWCY**C**R**TTYMPMSYLYGRGIQGPITDLVKS 293

TkOSC3 VLGVYEWEGCNPLPPEFWLFPSTFPFHPAE**MWCY**C**R**TTYMPMSYLYGKRIQGPLTPLVSS 286

AaOSC2 VLGVYEWEGCNPLPPEFWLFPEALPFHPAK**MWCY**C**R**TTYMPMSYLYGRKYHGPITDLVLQ 285

OeOSC VLGVYDWDGCNPLPPEFWLFPSFLPYHPAK**MWCY**C**R**TTYMPMSYLYGRKYHGPLTDLVLS 286

AaLUP VLGVYEWGGCNPMPPEFWLLPNFFPIHPGK**MMCY**G**R**LVYMPMSYLYAKRFVGKITKLVQE 286

TkLUP ILGVYEWGGCNPMPPEFWLMPKFFPIHPGK**MLCY**C**R**LVYMPMSYLYGKRFVGKITELVRD 286

ToLUP ILGVYEWGGCNPMPPEFWLMPKFFPIHPGK**MLCY**C**R**LVYMPMSYLYGKRFVGKITELVRD 286

TkOSC4 ALGLYEWSGSNPMPPEFWLLPSFLPISPGKI**WCY**C**R**MIYMPMSYLYGKRFIGPITPLILQ 287

AtLUP1 ILGVYDWSGTNPTPPELLMLPSFLPIHPGKI**LCY**S**R**MVSIPMSYLYGKRFVGPITPLILL 283

AtLUP2 ILGIYDWSGTNPMPPEIWLLPSFFPIHLGKT**LCY**T**R**MVYMPMSYLYGKRFVGPLTPLIML 286

SlOSC ILGAFEWIGTNPMPPEFWILPSFLPVHPAK**MWCY**C**R**TVYMPMSYLYGKRFVGPITPLILK 285

TkOSC5 ILGVCEWAGTNPMPPEFWILPSFLPMYPAKL**WCY**C**R**LVYMPMSYLYGKRFVGPITPLILQ 285

TkOSC6 ILGVCEWAGTNPMPPEFWLLPSFLPMCPAK**MWCY**C**R**LVYMPMSYLYGKRFVGPITPLVLQ 285

** :* * ** ***: ::*. :* .: ** * :******. * :* *:

TkOSC1 LRKEIHVIPYHQIDWNKQRHNCCKEDLYYPHTYIQDLLWDGLHYFSEPLVSKWPLKKL-R 352

TkOSC2 LRKEIHVIPYHQIDWNKQRHNCCKEDLYYPHTYIQDLLWDGLHYFSEPLITKWPFEKL-R 352

TkOSC3 LRKEIHLTPFEDINWNKQRNNCCKKDFYYPHSFLQDALWHSLHYLTEPVLKYWPFSKL-R 345

AaOSC2 LRQEIHPIPYHKINWNKQRHNCCKEDLYYPHSTVQDLLWDGLHYLSEPILKYWPFTKL-R 344

OeOSC LRNEIHIKPYNEIDWNKARHDCCKEDLYYPHSSIQDLLWDTLNYCAEPVMRRWPLNKI-R 345

AaLUP LRQELYTDPYHEINWNKARNNCAKEDLYYPHPLVQDILWGILHNVAEPILTHWPFSKL-R 345

TkLUP LRQELYTDPYDEINWNKARNTCAKEDLYYPHPFVQDMVWGVLHNVFEPVLTSRPLSTL-R 345

ToLUP LRQELYTDPYDEINWNKARNTCAKEDLYYPHPFVQDMVWGVLHNVVEPVLTSRPISTL-R 345

TkOSC4 LRKELYSIPYHEVNWRKVRHLCAKEDIYYPHPWLQDLSWDTLHLAVEPILTRWPFKSMIR 347

AtLUP1 LREELYLEPYEEINWKKSRRLYAKEDMYYAHPLVQDLLSDTLQNFVEPLLTRWPLNKLVR 343

AtLUP2 LRKELHLQPYEEINWNKARRLCAKEDMIYPHPLVQDLLWDTLHNFVEPILTNWPLKKLVR 346

SlOSC LREELYDQTYDEINWKKVRHVCAKEDLYYPHPFVQDLMWDSLYICTEPLLTRWPFNKL-R 344

TkOSC5 LRDELYLQPYNEINWKSIRHLCAKEDLYYPHPLLQDLMWDSLYICIEPLLNRWPLNKL-R 344

TkOSC6 LRDELYAQPYDEINWKSIRHLCAKEDLYYPHPFLQDLMWDSLYICTEPLLNRWPLNKL-R 344

**.*:: .: .::*.. *. .*:*: *.*. :** * **:: .*: .: *

TkOSC1 EKGLKRVLDLMQYNAEEGRYITLGCVEKSLQMMCFSALDPNGIDFKRHLARVPDYLWVAE 412

TkOSC2 AKGLKRVLDLMQYNAEEGRYITLGSVEKSLQMMCFYALDPNGIDFKRHLARVPDYLWVAE 412

TkOSC3 GRSLDRVVELMRYESEETRYMTIGCVEKSLQMMCWWAENPNGDEFKYHLARVPDYLWIAE 405

AaOSC2 ERGLKRAVELMRYGAEESRYMTIGCVEKSLQMMCWWAENPNGDEFKHHLARVPDYLWLAE 404

OeOSC QRALNKTIKYMRYGAEESRYITIGCVEKSLQMMCWWAHDPNGDEFKHHLARVPDYLWLAE 405

AaLUP EKAIKVAMEHVHYNDECSRYLGIGIVVKVLSLLATWVEDPNGDAYKRHLARISDYFWVAE 405

TkLUP EKALKVAMDHVHYEDKSSRYLCIGCVEKVLCLIATWVEDPNGDAYKRHLARIPDYFWVAE 405

ToLUP EKALKVAMDHVHYEDKSSRYLCIGCVEKVLCLIATWVEDPNGDAYKRHLARIPDYFWVAE 405

TkOSC4 EKALKTTMRHIHYEDENSRYITIGCVEKALCMLACWVEDPHGDYFKKHLSRIRDMIWVQE 407

AtLUP1 EKALQLTMKHIHYEDENSHYITIGCVEKVLCMLACWVENPNGDYFKKHLARIPDYMWVAE 403

AtLUP2 EKALRVAMEHIHYEDENSHYITIGCVEKVLCMLACWIENPNGDHFKKHLARIPDFMWVAE 406

SlOSC NKALEVTMKHIHYEDENSRYITMGCVEKVLSMLACWVEDPNGDHFKKHLARIPDFLWVAE 404

TkOSC5 QKALETTMKHIHYEDENSRYITIGSVVKVLCMLACWVEEPNGVCFKKHLARIPDYIWIAE 404

TkOSC6 QKALDTTMKHIHYEDENSRYITIGSVVKPLCMLACWVEDPNGVCFKKHIARIPDYIWVAE 404

..: .: :.* : .*: :* * * * ::. :*:* :* *::*: * :*: *

TkOSC1 DGMKMQSFGSQLWDCTLVTQAIIASDMVEEYGDSLKKANFYLKESQIKQNPKGDFENMCR 472

TkOSC2 DGMKMQSFGSQLWDCTLVTQAIIASDMVEEYGDSLKKANFYLKESQIKENPKGDFENMCR 472

TkOSC3 DGMTMHSFGSQVWDCSLATQAIIASNMVEEYDDCLEKAHFYLRESQVKENPSGDFTRMCR 465

AaOSC2 DGMTMHSFGSQLWDCVLATQAIIASDMVEEFGDSLKKAHFYIKESQIKQNPSGDFSKMCR 464

OeOSC DGMKMQSFGSQIWDSTLATQAVIATGMVEEYGDCLKKAHFYVKESQIKENPAGDFKSMYR 465

AaLUP DGLKIQSFGSQMWDAAFAIQAILSSNLAEEYGHVLKKAHDFVKSSQVRNNPPGDYSKMYR 465

TkLUP DGMKMQSFGCQMWDAAFAIQAILSSNLAEEYGPTLKKAHEFVKASQVRDNPPGDFSKMYR 465

ToLUP DGMKMQSFGCQMWDAAFAIQAIFSSNLTEEYGPTLKKAHEFVKASQVRDNPPGDFSKMYR 465

TkOSC4 DGMTVQSFGSQQWDSSLSVLALIDCNMIDETGSTLKKGHEFIKNSQVRDNPSGDFKSMYR 467

AtLUP1 DGMKMQSFGCQLWDTGFAIQALLASNLPDETDDALKRGHNYIKASQVRENPSGDFRSMYR 463

AtLUP2 DGLKMQSFGSQLWDTVFAIQALLACDLSDETDDVLRKGHSFIKKSQVRENPSGDFKSMYR 466

SlOSC DGMKMQGCGSQSWDASLAIQALLASEMNDEISDTLKNGHDFIKQSQVKDNPSGDFKVMYR 464

TkOSC5 DGMKMQSFGSQGWDASLAIQALLATDLTDEIGSTLMKGHKFVKASQVKDNPSGYFKNMHR 464

TkOSC6 DGMKMQSFGSQKWDAGFAIQALLAADLTEENGSTLMKGHEFIKASQVKDNPSGDFKSMHR 464

**:.::. *.* ** : *:: : :* . * ..: ::. **:.:** * : * *

TkOSC1 QFTKGAWTFSDQDQGWVVSDCTAEAVKCLLALSQMPQEISGEKVDVERLYDAINVLLYL**Q** 532

TkOSC2 QFTKGAWTFTDQDQGWVVSDCTAEAVKCLLAMSQMPQEIAGEKVEVERLYDAINVLLYL**Q** 532

TkOSC3 QFTKGSWTFSDQDHGWTVSDCTAEALKCLLLLSNMPKNIAGEKDDTARLYEAVNVLLYM**Q** 525

AaOSC2 QFTKGAWTFSDQDQGWVVSDCTAEALKCLLLLSQMPEEISGKKADNERLYEAVNVLLYL**Q** 524

OeOSC HFTKGAWTFSDQDQGWVVSDCTAEALKCLLLLSQLPTETAGEKADVERLYEAVNVLLYL**Q** 525

AaLUP HTSKGAWTFSMQDNGLQVSDCTAEGLKVALMYSQISPELVGEKLETERLYDAVNVILSL**Q** 525

TkLUP HTSKGAWTFSIQDHGWQVSDCTAEGLKVALLYSQMSPELVGEKLETEHLYDAVNVILSL**Q** 525

ToLUP HTSKGAWTFSIQDHGWQVSDCTAEGLKVSLLYSQMNPKLVGEKVETEHLYDAVNVILSL**Q** 525

TkOSC4 HISKGGWTYSDQDHGWQVSDCTAHGLMSCLLLSKMPLEIVGEKMETERLFDCINLLLSL**Q** 527

AtLUP1 HISKGAWTFSDRDHGWQVSDCTAEALKCCLLLSMMSADIVGQKIDDEQLYDSVNLLLSL**Q** 523

AtLUP2 HISKGAWTLSDRDHGWQVSDCTAEALKCCMLLSMMPAEVVGQKIDPEQLYDSVNLLLSL**Q** 526

SlOSC HISKGSWAFADQDLGWQVSDCTAEALKCCLLFSTMPPEIVGEAMDPVRLYDSVNVILSL**Q** 524

TkOSC5 HISKGSWSFSDQDHGWQGSDTTAEALKCCLLFSTMPPEIVGEKMKPEQLNDAVNVILSL**Q** 524

TkOSC6 HISKGSWTFSDQDHGWQISDCTAEGLKCCLLFLTMPAEIVGEHMKPEQFNDAVNVILSL**Q** 524

: :**.*: : .* * ** ** .: : : . *: . .: :.:*::* :*

TkOSC1 SPET**G**GFAIWEAPVPKPYLEKLNPSELFADIVVEREHVECTGSIIQTLQTFKTLHPGHRQ 592

TkOSC2 SPET**G**GFAVWEVPVPKPYLEKLNPSELFADITVEREHVECTGSIIQALQTFKTLHPGHRE 592

TkOSC3 SPVS**G**GFAVWEPPIPKPFLQLLNPSEIFADIVVEKEHVETTSSIIGALIEFKRVHPNHRK 585

AaOSC2 SPIS**G**GFAIWEPPVPQPYLQMLNPSEIFADIVVEKEHVECTSSIIKALLAFKDLHPGHRE 584

OeOSC SPES**G**GFAIWEPPVPQPYLQMLNPSEIFADIVVETEHVECSASIIQALLAFKRLYPGHRE 585

AaLUP SVN-**G**GFPAWEPQRAYSWLEKFNPTEFFEDTLIEREYTECTSSAIQALTLFMKLHPGHRT 584

TkLUP SEN-**G**GFPAWEPQRAYAWLEKFNPTEFFEDVLIEREYVECTSSAIQGLTLFKKLHPGHRT 584

ToLUP SEN-**G**GFPAWEPQRAYAWLEKFNPTEFFEDVLIEREYVECTSSAIQGLTLFKKLHPGHRT 584

TkOSC4 YKN-**G**GFSGWEPAGAPKWLEMLNPSEMFADIMIEIQYVECTSSALQALILFKKLYPEHRS 586

AtLUP1 SGN-**G**GVNAWEPSRAYKWLELLNPTEFMANTMVEREFVECTSSVIQALDLFRKLYPDHRK 582

AtLUP2 GEK-**G**GLTAWEPVRAQEWLELLNPTDFFTCVMAEREYVECTSAVIQALVLFKQLYPDHRT 585

SlOSC SKN-**G**GLSAWEPAGAPEYLELLNPTEFFEDIVIEHEHVECTSSAIQALVRFKKLYPGHRT 583

TkOSC5 SKN-**G**GLASWEPAGSSEWLEIFNPTEFFADIVIEHEYVECTSSAIQAIVLFNKLYPQHRK 583

TkOSC6 SKN-**G**GLAAWEPAGSSEWLEVLNPTEFFADIVIEHEYVECTSSASQALVLFKKLYPGHRR 583

**. ** . :*: :**:::: * :..* :.: : * ::* **

TkOSC1 KEIEVAIEKGIRFLENR**Q**QEN**G**S**W**YGYWGICYLYGTYFVLQGLVACGQTYENSEAVRKAV 652

TkOSC2 KEIEVAIEKGIHFLENR**Q**QEN**G**S**W**YGFWGICYIYGTYFVLQGLVACGKTYENCEAVRKAV 652

TkOSC3 EEIEYSISNGIRYLEET**Q**WHD**G**S**W**YGYWGVCFIYGTFFALRALSTAGKTYKNNEAACKGV 645

AaOSC2 KEIEISVAKAVCFLEGK**Q**CHD**G**S**W**YGYWGICFLYGTFFTLAGLVSAGKTYDNSEAVRKAV 644

OeOSC KEIEISVAKAISFLEGR**Q**WPD**G**S**W**YGYWGICFLYGTFFVLGGLSAAGKTYENSEAVRKGV 645

AaLUP MEIEDCISRAVNYIKDA**Q**NPD**G**S**W**YGYWGICYTYGAWFAVDALVACGYNYHNSPAIQKSC 644

TkLUP KEIEHCISRAIKYVEDT**Q**ESD**G**S**W**YGCWGICYTYGTWFAVDALVACGKNYHNSPALQKAC 644

ToLUP KEIEHCISRAVKYVEDT**Q**ESD**G**S**W**YGCWGICYTYGTWFAVDALVACGKNYHNCPALQKAC 644

TkOSC4 QEVANCISNAIRFLEDT**Q**WPD**G**S**W**YGEWGVCFTYATWFATKGLAAAGKTYEQSPTIRKAT 646

AtLUP1 KEINRSIEKAVQFIQDN**Q**TPD**G**S**W**YGNWGVCFIYATWFALGGLAAAGETYNDCLAMRNGV 642

AtLUP2 KEIIKSIEKGVQFIESK**Q**TPD**G**S**W**HGNWGICFIYATWFALSGLAAAGKTYKSCLAVRKGV 645

SlOSC TEVDNFINNGVKYIEDV**Q**EPD**G**S**W**YGNWGVCFIYASWFALGGLAAVGLSYSNCAAVRKSV 643

TkOSC5 KEIETFLTGASGYLEKL**Q**TKD**G**S**W**YGNWGVCFTYGTWFGIGGLTAVGKTFENCQAIQKAV 643

TkOSC6 KEIESFLTGASGYLEKI**Q**MED**G**S**W**YGNWGVCFTYGTWFALGGLTAVGKTFENCLAIRKAV 643

*: : . ::: * :***:* **:*: *.::* .* : * .: . : :.

TkOSC1 KFFLST**Q**NSE**G**G**W**GENFESCPQEKFIPLEGNRTNFVQTSWAMLGLLCGGQAERDPTPLHK 712

TkOSC2 KFFLST**Q**NSE**G**G**W**GEHFESCPQEKFIPLEGNRTHFVHTSWAMLGLLYGGQVERDPTPLHK 712

TkOSC3 KFLLSI**Q**NEE**G**G**W**GESLLSCPTEVYTPLDGNQTNLVQTSWAMLGLLFGGQVDRDPTPLHR 705

AaOSC2 NFFLST**Q**NEE**G**G**W**GESIKSCPSEVYTPLDGNRTNLVQTSWAMLGLMFGGQAERDPTPLHK 704

OeOSC NFLLST**Q**NEE**G**G**W**GECLESCPSMKYTPLEGNRTNLVQTSWAMLGLMYGGQAERDPTSLHK 705

AaLUP EFLLSK**Q**LSD**G**G**W**GESYLSSANKTYTNLDGNRSNLVQTSWALISLIKAGQAEIDLTPISN 704

TkLUP KFLLSK**Q**LPD**G**G**W**GESYLSSSNKVYTNLEGNRSNLVHTSWALISLIKAGQAEIDPTPISN 704

ToLUP KFLLSK**Q**LPD**G**G**W**GESYLSSSNKVYTNLEGNRSNLVHTSWALISLIKAGQAEIDPTPISN 704

TkOSC4 EFLLKH**Q**QED**G**G**W**GESYLSCPNLEFTSLEESRSNVVQTSWCLMSLIQCGQAERDLTPIHR 706

AtLUP1 HFLLTT**Q**RDD**G**G**W**GESYLSCSEQRYIPSEGERSNLVQTSWAMMALIHTGQAERDLIPLHR 702

AtLUP2 DFLLAI**Q**EED**G**G**W**GESHLSCPEQRYIPLEGNRSNLVQTAWAMMGLIHAGQAERDPTPLHR 705

SlOSC EFLLRT**Q**RSD**G**G**W**GESYRSCPDKVYRELETEHSNLVQTAWALMGLIHSGQVERDPRPLHR 703

TkOSC5 KFLLET**Q**LED**G**G**W**GESYKSCPEKIYIPLEGGRSNLVHTSWAMIGLIQSQWMERDSTPIHK 703

TkOSC6 KFLLET**Q**LED**G**G**W**GESYKSCPEKRYVPLEEGRSNLVHTAWGMMGLIHSQQMERDPMPLHK 703

*:* * :***** *.. : : .::.*:*:* ::.*: . : * .: .

TkOSC1 AAKLLING**Q**MDN**G**DFPQQEITGVYMKNCMLHYAEYRNTFPLWALGEYRKRVWLAKQET-- 770

TkOSC2 ASKLLING**Q**TDN**G**DFPQQEITGVYNKNCMLHYAEYRNTFPLWALGEYRKRVWLAKQET-- 770

TkOSC3 AAKLLINA**Q**MDN**G**DFPQQEITGVYMKNCLLLYAQYRNIFPLWALGEYRKRVWSIKKGIQN 765

AaOSC2 AAKILINA**Q**MDD**G**DFPQQEITGVYMKNCMLHYAEYRNIFPLWALGEYRKRVWVKTK---- 760

OeOSC AAKLLIDA**Q**MDD**G**DFPQQEITGVYMKNCMLHYAQYRNIFPLWALGEYRKRVWSSQSL--- 762

AaLUP GIRLLINS**Q**MKE**G**DFPQQDITGVFMRNCTLNYSAYRNIFPIWALGEYRRLISN------- 757

TkLUP GVRLLINS**Q**MEE**G**DFPQQEITGVFMKNCNLNYSSYRNIFPIWALGEYRRIVQNI------ 758

ToLUP GVRLLINS**Q**MEE**G**DFPQQEITGVFMKNCNLNYSSFRNIFPIWALGEYRRIVQNI------ 758

TkOSC4 GAKFLINS**Q**MED**G**DFPQKEITGSFRKNCMLHYACHRNIFPMWALAEYKNRVSKII----- 761

AtLUP1 AAKLIINS**Q**LEN**G**DFPQQEIVGAFMNTCMLHYATYRNTFPLWALAEYRKVVFIVN----- 757

AtLUP2 AAKLIITS**Q**LEN**G**DFPQQEILGVFMNTCMLHYATYRNIFPLWALAEYRKAAFATHQDL-- 763

SlOSC AAKLLINF**Q**MED**G**DFPQQEITGVFLRNCMMHYALYRNIFPLWGLAEYRRNVLVPLKHNYI 763

TkOSC5 AARLLINS**Q**LEN**G**DFPQQEITGVFMKNCMLHYALYRNIYPMWALADYRKKVLSTCKK--- 760

TkOSC6 AAKLLINS**Q**LEN**G**DFPQQKIAGVFKMNCMLHYALYRNIFPMWALADYRKHVLKGI----- 758

. .::* * .:*****:.* * : .* : *: .** :*:*.*.:*..

**(b)**

TkCYP716D60 ---MILFVALTLLILI--VSYLLTFLYWNRKCG-RKINL**PPG**SF**G**W**P**FIGESLALLRAGW 54

AaCYP716D22 ---MIQVLTSIFLFLV--IPYILWTLYTKSSQR-SKINL**PPG**SF**G**W**P**FLGESLSLLRACW 54

TkCYP716A263 ---MAMFYASLFSLFTIVVSCLLRFAFFKSKSA-ANINL**PPG**RR**G**W**P**VIGETVEFVTAGW 56

AaCYP716A14v2 MVDLILIYSIFLYLIVVLVPLSLYFVFYKSKPV-VDRKL**PPG**QT**G**W**P**VIGETLEFLTNGW 59

AtCYP716A1 -----MYMAIMIILFLSSILLSLLLLLRKHLSHFSYPNL**PPG**NT**G**L**P**LIGESFSFLSAGR 55

MtCYP716A12 --MEPNFYLSLLLLFVSFISLSLFFIFYKQKSP---LNL**PPG**KM**G**Y**P**IIGESLEFLSTGW 55

VvCYP716A15 ---MEVFFLSLLLIFVLSVSIGLHLLFYKHRSHFTGPNL**PPG**KI**G**W**P**MVGESLEFLSTGW 57

BpCYP716A180 ---MEHFYLSLLLLFVSFVTLSLFTLFYKHRSHFTGPNL**PPG**KT**G**Y**P**MIGESLEFLSTGW 57

: :: : * : :**** * *.:**:. :: .

TkCYP716D60 EGKPERFIRERVEKHGSP--LIFKTSPLGDRIAVLCGPAGNKFLFGNENKLVAAWWPTRV 112

AaCYP716D22 AGEPERFINERIEKHGNS--VVFKTSLLGDYMAVLCGPAGNKFLFGNENKLVAAWWPLPV 112

TkCYP716A263 KGHPEKFIVDRMKKFSHH---VFRTSLMLEDAAVFCGPEGNKFLFSNENKLVRFWLPASA 113

AaCYP716A14v2 KGHPEKFIFDRMARFSPH---VFKTSLMLEDAAVFCGSAGNKFLFSNENKLVKAWWPASV 116

AtCYP716A1 QGHPEKFITDRVRRFSSSSSCVFKTHLFGSPTAVVTGASGNKFLFTNENKLVVSWWPDSV 115

MtCYP716A12 KGHPEKFIFDRMRKYSSE---LFKTSIVGESTVVCCGAASNKFLFSNENKLVTAWWPDSV 112

VvCYP716A15 KGHPEKFIFDRISKYSSE---VFKTSLLGEPAAVFAGAAGNKFLFSNENKLVHAWWPSSV 114

BpCYP716A180 KGHPEKFIFDRMTKFSSE---VFKTSLLGQPAAVFCGAACNKFLFSNENKLVTAWWPDSV 114

* **.** :*: ... :*.* . . .* *. ***** ****** * * .

TkCYP716D60 RKLFGRCLITI-RGDEAKLMRKMLLSYLGPDAFASHNSATMDIVTRRHIDVHWRGKEEVN 171

AaCYP716D22 RKLFGRCLITI-RGDEAKWMRKMLLSYLGPDAFATHYAVTMDVVTRRHIEVHWRGKEEVN 171

TkCYP716A263 RKILPFTQYCG------DDIAKMVRYHLKPETI-RQYVPIIDMVAQKHFETRWEGKDQIL 166

AaCYP716A14v2 EKILPSAKETT-------NQRKMLSRSFRPESL-RQYVPVMDMMAQRHFKTEWDGMDQIV 168

AtCYP716A1 NKIFPSSMQTS-SKEEARKLRMLLSQFMKPEAL-RRYVGVMDEIAQRHFETEWANQDQVI 173

MtCYP716A12 NKIFPTTSLDSNLKEESIKMRKLLPQFFKPEAL-QRYVGVMDVIAQRHFVTHWDNKNEIT 171

VvCYP716A15 DKVFPSSTQTS-SKEEAKKMRKLLPQFFKPEAL-QRYIGIMDHIAQRHFADSWDNRDEVI 172

BpCYP716A180 NKIFPSSTQTSNSKEEAKKMRKLLPQFLKPEAL-QKYISIMDTIAQRHFASGWEGQKEVT 173

*:: :: : *::: . :* ::..*: * . .::

TkCYP716D60 VFKTVKLYAFELACRLFLSLEEPNHIEKLGALFNVFLKGIIELPIDFPGTRFHSSKKAAA 231

AaCYP716D22 VYQTVKLYAFELACRLFMSLEEPNHIAKLASLFNVFLKGIIELPIDFPGTRFYSSKKAAA 231

TkCYP716A263 THETTKNFTFLVACKIFFSIDEPEWVNNLSGPFKRLAPGLFSIPINLPGTPFRRAINART 226

AaCYP716A14v2 THEVTQNFTFSLACKIFVSIEDPEEVKHLSGPFEKFAPGIFSIPIDLPWTPLRRAIHAGN 228

AtCYP716A1 VFPLTKKFTFSIACRSFLSMEDPARVRQLEEQFNTVAVGIFSIPIDLPGTRFNRAIKASR 233

MtCYP716A12 VYPLAKRYTFLLACRLFMSVEDENHVAKFSDPFQLIAAGIISLPIDLPGTPFNKAIKASN 231

VvCYP716A15 VFPLAKRFTFWLACRLFMSIEDPAHVAKFEKPFHVLASGLITVPIDLPGTPFHRAIKASN 232

BpCYP716A180 VFPLAKRYTFWLACRLFLSLEDPNHIARFADPFHSVASGIISIPIDLPGTPFNRGIKASN 233

.. .: ::* :**. *.*::: : .: * . *:: :**::* * : . :*

TkCYP716D60 AIRTEVMAIIKERRIALEEGKASSSQDLLSHLLTSSDENGKFLTATEIANNILLLLF**AG**H 291

AaCYP716D22 AIRTELKTLINARRVALAEGKATSSQDLLSHLLTSSDEDGKYLTEMEIANNILLLLF**AG**H 291

TkCYP716A263 FIGKELTAIVKQRKIDLANGKASPTQDILSLLL--GDDYGKYMADTAIADLIIGLMI**GG**Y 284

AaCYP716A14v2 FIRKEIIAIIKQRKIDLADGKASPTQDILSQML--CDEESQNIAEADIADVIIGLLI**GG**H 286

AtCYP716A1 LLRKEVSAIVRQRKEELKAGKALEEHDILSHMLMNIGET----KDEDLADKIIGLLI**GG**H 289

MtCYP716A12 FIRKELIKIIKQRRIDLAEGTASPTQDILSHMLLTSDENGKSMNELNIADKILGLLI**GG**H 291

VvCYP716A15 FIRKELRAIIKQRKIDLAEGKASQNQDILSHMLLATDEDGCHMNEMEIADKILGLLI**GG**H 292

BpCYP716A180 FIRKELSLIIKQRRVDLGEGKASPTQDILSHMLLTSDESGQYMTELDIADKILGLLI**GG**H 293

: .*: ::. *. * *.* :*:** :* .: :*: *: *::.*:

TkCYP716D60 **DT**STVTITLVMKSLGEYPDVYEKVLREQLEISKGKEVGEMLKWDDVQKMRYSWNVVS**E**VM 351

AaCYP716D22 **DT**STCSITLLMKRLAEHPAVYDKVLKEHLDICKTKEAGELLKWEDIQKMKYSWNVVS**E**VM 351

TkCYP716A263 **D**STSSTCTFIVNYLAELPEIYEKVYKEQTEIAKSKTSKELLNWDDISKMKYSWNVVS**E**VL 344

AaCYP716A14v2 **D**NASSTCAFIVKFLADLPEIYEGVLKEQLEIAKSKAPGELLNWEDLSKMKYSWNVAC**E**VL 346

AtCYP716A1 **DT**ASIVCTFVVNYLAEFPHVYQRVLQEQKEILKEKKEKEGLRWEDIEKMRYSWNVAC**E**VM 349

MtCYP716A12 **DT**ASVACTFLVKYLGELPHIYDKVYQEQMEIAKSKPAGELLNWDDLKKMKYSWNVAC**E**VM 351

VvCYP716A15 **DT**ASAAITFLIKYMAELPHIYEKVYEEQMEIANSKAPGELLNWDDVQNMRYSWNVAC**E**VM 352

BpCYP716A180 **DT**ASAACTFIVKYLAELPHIYEGVYNEQMEIANSKAPGELLNWEDIQKMRYSWNVAC**E**VL 353

*.:: ::::: :.: * :*: * *: :* : * * *.*:*:.:*.*****..**:

TkCYP716D60 **R**ITPPVLGSFREALVDFEYAGYTIPKGWKLYWSAVSTHKDEANFQHATQFDPS**R**FEGTGP 411

AaCYP716D22 **R**LTPPIVGAYREALVDFEYGGYTIPKGWKLYWSAAAAHKDEANFEDVERFDPS**R**FEGAGP 411

TkCYP716A263 **R**LVPPTQGTFREAICNFTFRQYSIPKGWKLYWSTNSTHKNPNFFLEPEKFDPS**R**FDSQAL 404

AaCYP716A14v2 **R**LAPPLQGSFREAMTDFVYNGYSIPKGWKLYWSALSTHKNPEVFTEPQKLDPS**R**FDGKGP 406

AtCYP716A1 **R**IVPPLSGTFREAIDHFSFKGFYIPKGWKLYWSATATHMNPDYFPEPERFEPN**R**FEGSGP 409

MtCYP716A12 **R**LSPPLQGGFREAITDFMFNGFSIPKGWKLYWSANSTHKNAECFPMPEKFDPT**R**FEGNGP 411

VvCYP716A15 **R**LAPPLQGAFREAITDFVFNGFSIPKGWKLYWSANSTHKSPECFPQPENFDPT**R**FEGNGP 412

BpCYP716A180 **R**LAPPLQGAFREAINDFIFNGFSIPKGWKLYWSANSTHRSAEYFPEPEKFDPS**R**FEGRGP 413

*: ** * :***: * : : **********: ::* . * .::*.**:. .

TkCYP716D60 TPFTYVP**F**GG**G**PRM**C**L**G**KEFSRMEVLVFLHNIVTNFKWDLLIPDE-KIEYDPM-ATPVKG 469

AaCYP716D22 TPFTYVP**F**GG**G**PRM**C**L**G**KEFSRLEVLVYLHNIVTNFKWDLLIPDE-KIEYDPM-AVPEKG 469

TkCYP716A263 VPYTYVP**F**GG**G**CHL**C**P**G**KEFARVELLVFIHHLVRKFKLKKIFSKE-DIIFDLLQPKLAKG 463

AaCYP716A14v2 APYTFVP**F**GG**G**PHM**C**P**G**REYARLEILVFMHHLVIKYKWEKVIPNE-QIIVNPM-PKLAKG 464

AtCYP716A1 KPYTYVP**F**GG**G**PRM**C**P**G**KEYARLEILIFMHNLVNRFKWEKVFPNENKIVVDPL-PIPDKG 468

MtCYP716A12 APYTFVP**F**GG**G**PRM**C**P**G**KEYARLEILVFMHNLVKRFKWEKVIPDE-KIIVDPF-PIPAKD 469

VvCYP716A15 APYTFVP**F**GG**G**PRM**C**P**G**KEYARLEILVFMHNVVKRFKWDKLLPDE-KIIVDPM-PMPAKG 470

BpCYP716A180 APYTFVP**F**GG**G**PRM**C**P**G**KEYARLEILVFMHNLVKRFRWEKMIPDE-KIVVDPM-PMPAKG 471

*:*:****** .:* *.*::*:*:*:::*::* .:. . ::..* .* : : . *.

TkCYP716D60 LPIRVHPHHV 479

AaCYP716D22 LPIRLHPHQV 479

TkCYP716A263 LPIHLYPHKL 473

AaCYP716A14v2 LPLRLYPRYA 474

AtCYP716A1 LPIRIFPQS- 477

MtCYP716A12 LPIRLYPHKA 479

VvCYP716A15 LPVRLHPHKP 480

BpCYP716A180 LPVRLYPHKA 481

**:.:.*.

**Supplementary Figure S3.** **Heterologous expression of *T. koksaghyz* *OSC* and *CYP716D60* in *N. benthamiana*.** (a) β-amyrin could not be detected using SIM for m/z 218 in tobacco extracts by coexpression of *Tkhmgrc1* and *TkLUP*. (b) No TkOSC2 or TkOSC5 products could be detected by coexpression of *TkOSC2* or *TkOSC5* with *Tkhmgrc1*, respectively. No CYP716D60 products could be detected by coexpression with (c) *Tkhmgrc1* and *TkLUP* or (d) *Tkhmgrc1* and *TkOSC1*.


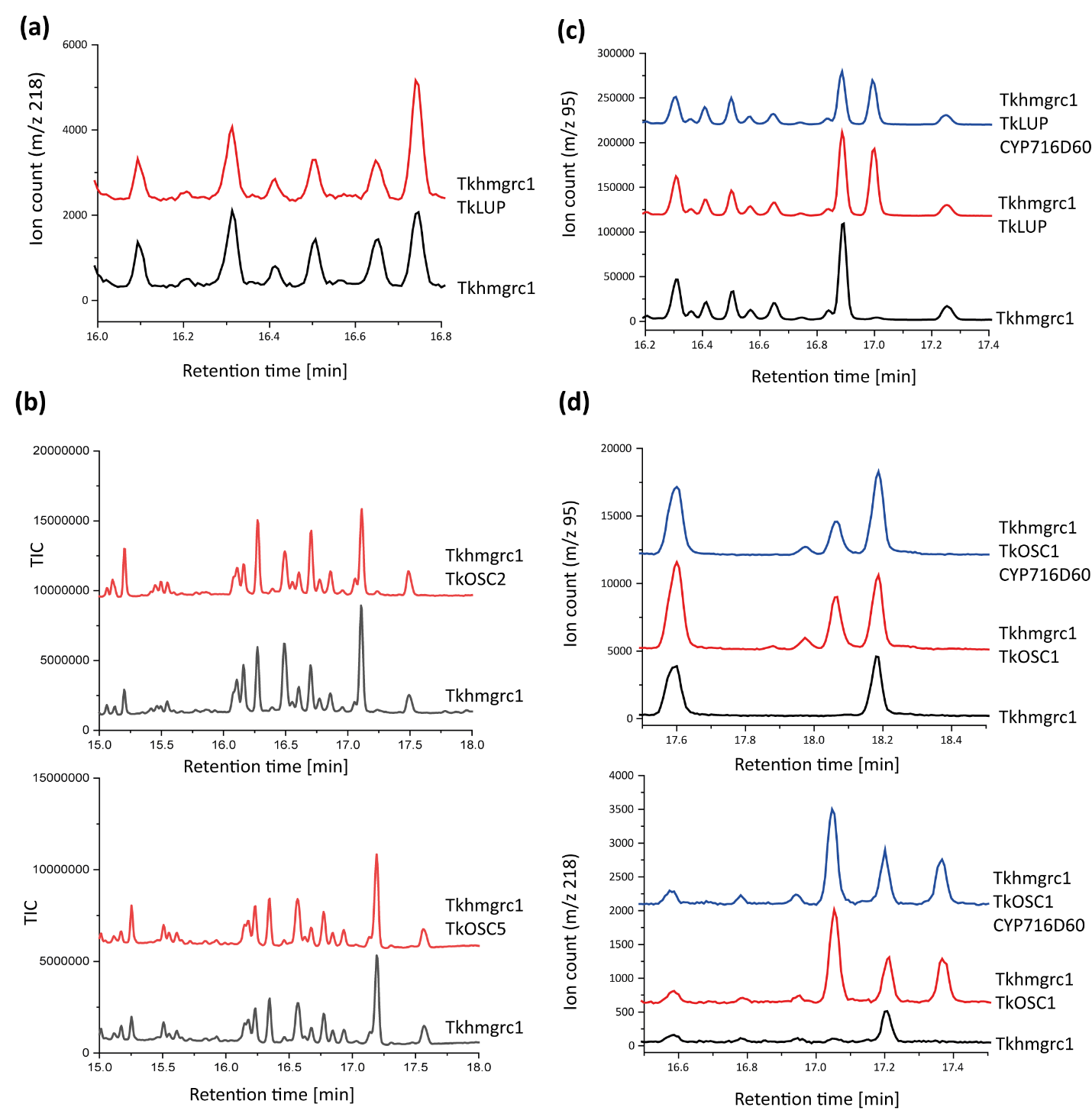


**Supplementary Figure S4.** **Heterologous expression of *T. koksaghyz* *TkOSC6* and *CYP716A263* in *N. benthamiana* and *S. cerevisiae*.** Selected ion monitoring (SIM) GC-chromatograms of leaf extracts of tobacco plants infiltrated with *Tkhmgrc1* and *TkOSC6* and *Tkhmgrc1*, *TkOSC6* and *CYP716A263* (a) and SIM GC-chromatograms of yeast extracts expressing *TkOSC6* and *TkOSC6* and *CYP716A263/AtR2* (b). Mass spectra of putative triterpene compounds at specific retention times (Rt) are indicated. β-amyrin as an OSC6 product could be clearly identified by mass spectra in tobacco as well as in yeast extracts. α-amyrin as an OSC6 product and β-amyrone as a CYP716A263 product could be clearly identified by mass spectra in tobacco extracts.

**
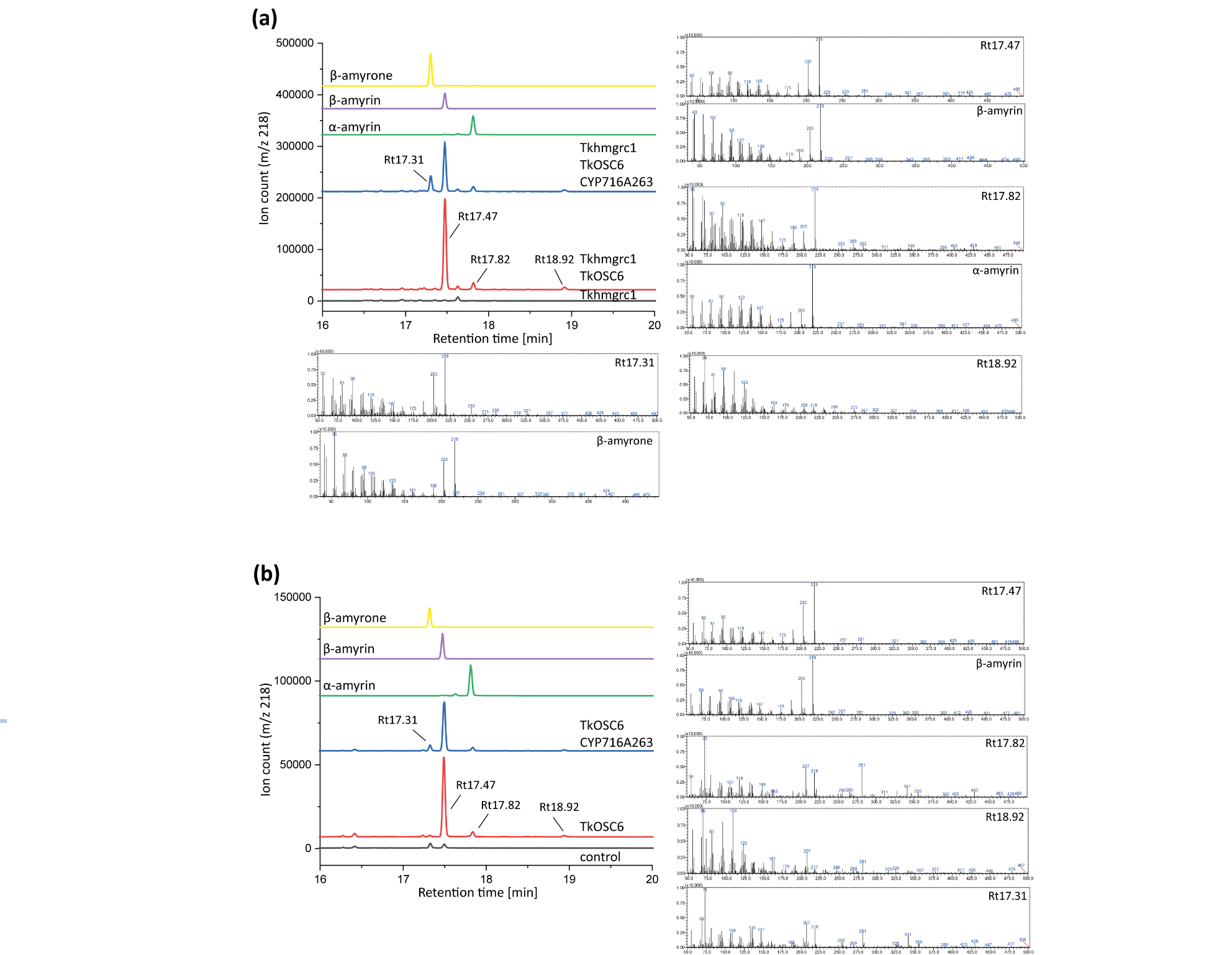
**

**Supplementary Figure S5.** **Heterologous expression of *T. koksaghyz* *TkOSC1* in *S. cerevisiae*.** Four additional unidentified products could be detected for TkOSC1. The mass spectra of three of these products matched with so far unknown triterpenes that could also be detected in HPLC fractions of the NR acetone extract: Mass spectra of Rt 17.63 min and Rt 17.99 min corresponds to compound 1 and 3 in fraction 2, respectively, and mass spectrum of Rt 18.85 min corresponds to compound 4 in fraction 5 (Fig. S1).

**
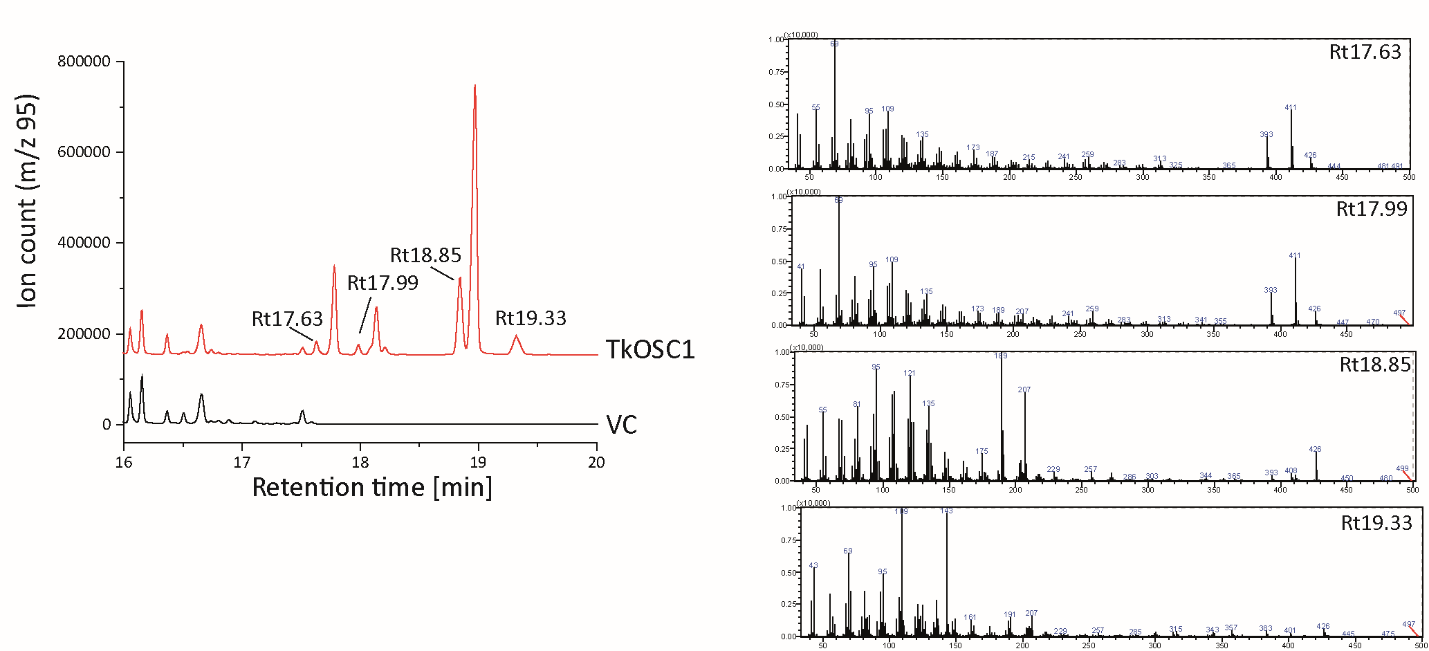
**

**Supplementary Table S1. NMR data of lup-19(21)-en-3-ol and lup-19(21)-en-3-one.**

**Lup-19(21)-en-3-ol**

| **Position** | **C [ppm]** | **H [ppm]** | **Form** | **Integral** | **J-Coupling Constant [Hz]** | **COSY** | **HMBC** | **HSQC** | **NOESY** |
| --- | --- | --- | --- | --- | --- | --- | --- | --- | --- |
| **1a** | 38.63 | 0.95 | m | 1 | - | 2 | 25 | + | - |
| **1b** | 38.63 | 1.69 | m | 1 | - |  | 3,25 | + | - |
| **2** | 27.48 | 1.63 | m | 2 | - | 1, 3 | 3 | + | - |
| **3** | 79.17 | 3.20 | dd | 1 | 11.0;5.0 | 2 | 1,2,23,24 | + | 5,23 |
| **4** | 38.88 | - | - | - | - | - | 23,24 | - | - |
| **5** | 55.19 | 0.71 | m | 1 | - | 6 | 6,23,24,25 | + | 3,9,29 |
| **6a** | 18.37 | 1.40 | m | 1 | - | 5 | 5,7 | + | - |
| **6b** | 18.37 | 1.54 | m | 1 | - |  | 5,7 | + | - |
| **7** | 34.14 | 1.40 | m | 2 | - | - | 6 | + | - |
| **8** | 41.01 | - | - | - | - | - | 7,26,27 | - | - |
| **9** | 50.06 | 1.37 | m | 1 | - | - | 7,25,26 | + | 5 |
| **10** | 37.10 | - | - | - | - | - | 25 | - | - |
| **11a** | 20.94 | 1.00 | m | 1 | - | - | 12 | + | - |
| **11b** | 20.94 | 1.51 | m | 1 | - | 12 | 12 | + | - |
| **12a** | 25.75 | 1.35 | m | 1 | - | 11 | - | + | 29 |
| **12b** | 25.75 | 1.95 | m | 1 | - | - | - | + | 20 |
| **13** | 35.28 | 1.98 | m | 1 | - | 18 | 12 | + | 12a,28,30 |
| **14** | 43.36 | - | - | - | - | - | 26,27 | - | - |
| **15a** | 27.48 | 1.01 | m | 1 | - | - |  | + | - |
| **15b** | 27.48 | 1.72 | dd | 1 | 13.1;4.6 | - | 27 | + | - |
| **16a** | 33.66 | 1.47 | ddd | 1 | 12.9,4.4,2.4 | - | 15,28 | + | - |
| **16b** | 33.66 | 1.61 | m | 1 |  |  | 15,17,28 | + | - |
| **17** | 46.01 | - | - | - | - | - | 16,28 | - | - |
| **18** | 52.84 | 2.16 | dd | 1 | 11.6; 1.9 | 13 | 28 | + | 29 |
| **19** | 155.03 | - | - | - | - | - | 22,29,30 | - | - |
| **20** | 29.11 | 2.39 | m | 1 | 6.5 | 29,30 | 21,29,30 | + | 12b,29,30 |
| **21** | 120.34 | 5.33 | m | 1 | - | 22 | 20,22 | + | 22,30 |
| **22** | 45.28 | 1.82 | d | 2 | 2.2 | 21 | 20,28 | + | 21 |
| **23** | 27.98 | 0.97 | s | 3 | - | - | 3,4,5,24 | + | 3 |
| **24** | 15.38 | 0.77 | s | 3 | - | - | 3,4,5,23 | + | - |
| **25** | 16.01 | 0.85 | s | 3 | - | - | 4,5,9,10 | + | - |
| **26** | 15.92 | 1.05 | s | 3 | - | - | 7,8,9,14 | + | - |
| **27** | 14.42 | 0.98 | s | 3 | - | - | 8,13,14,15 | + | - |
| **28** | 19.15 | 0.82 | s | 3 | - | - | 16,17,18,22 | + | - |
| **29** | 22.29 | 0.99 | d |  | 6.6 | 20 | 19,20,30 | + | 13,18 |
| **30** | 22.58 | 1.01 | d |  | 6.6 | 20 | 19,20,29 | + | 15b |

L**up-19(21)-en-3-one**

| **Position** | **C [ppm]** | **H [ppm]** | **Form** | **Integral** | **J-Coupling Constant [Hz]** | **COSY** | **HMBC** | **HSQC** |
| --- | --- | --- | --- | --- | --- | --- | --- | --- |
| **1a** | 39.63 | 1.42 | m | 1 |  | 2b | 7 | + |
| **1b** | 39.63 | 1.91 | ddd | 1 | 2.8;12.2 | 2b | 2,10 | + |
| **2a** | 34.31 | 2.42 | ddd | 1 | 4.3;7.7;14.9 | - | 1 | + |
| **2b** | 34.31 | 2.49 | ddd | 1 | 2.3;9.9;15.6 | 1a | - | + |
| **3** | 218.46 | - | - | - | - | - | 23,24 | - |
| **4** | 47.49 | - | - | - | - | - | 23,24 | - |
| **5** | 54.91 | 1.33 | dd | 1 | 2.9;11.8 | - | 23,24,25 | + |
| **6a** | 19.79 | 1.03 | m | 1 | - | 7 | - | + |
| **6b** | 19.79 | 1.47 | m | 1 | - | 7 | - | + |
| **7a** | 33.50 | 1.43 | d | 1 | 9.0 | 6 | 26 | + |
| **7b** | 33.50 | 1.48 | m | 1 | - | 6 | 26 | + |
| **8** | 41.02 | - | - | - | - | - | 26,27 | - |
| **9** | 49.53 | 1.47 | m | 1 | - | 11 | 25,26 | + |
| **10** | 36.87 | - | - | - | - | - | 1,25 | - |
| **11a** | 21.50 | 1.38 | m | 1 | - | 9,12 | - | + |
| **11b** | 21.50 | 1.51 | m | 1 | - | 9,12 | - | + |
| **12a** | 25.79 | 1.36 | m | 1 | - | 11 | - | + |
| **12b** | 25.79 | 1.96 | m | 1 | - | 11 | - | + |
| **13** | 35.50 | 1.99 | m | 1 | - | - | 27 | + |
| **14** | 43.55 | - | - | - | - | - | 26,27 | - |
| **15a** | 27.50 | 1.74 | td | 1 | 4.7;13.2 | - | 27 | + |
| **15b** | 27.50 | 2.0 | m | 1 |  | - | 27 | + |
| **16** | 33.69 | 1.64 | td | 2 | 3.9;8.6 | - | 28 | + |
| **17** | 46.07 | - | - |  |  | - | 28 | - |
| **18** | 52.82 | 2.16 | d | 1 | 11.5 | - | 28 | + |
| **19** | 154.99 | - | - |  | - | - | 29,30 | + |
| **20** | 29.22 | 2.39 | dd | 1 | 3.7;7.6 | 29,30 | 29,30 | + |
| **21** | 120.54 | 5.36 | m | 1 | - | 18,22 | - | + |
| **22** | 45.31 | 1.86 | d | 2 | 1.6 | 21 | 28 | + |
| **23** | 26.64 | 1.07 | s | 3 | - | - | 3,4,24 | + |
| **24** | 21.17 | 1.03 | s | 3 | - | - | 3,4,23 | + |
| **25** | 15.99 | 0.94 | s | 3 | - | - | 5,10 | + |
| **26** | 15.80 | 1.08 | s | 3 | - | - | 7,8,14 | - |
| **27** | 14.51 | 0.99 | s | 3 | - | - | 13,14,15 | + |
| **28** | 19.22 | 0.82 | s | 3 | - | - | 16,17,18,22 | + |
| **29** | 22.41 | 1.01 | d | 3 | 6.7 | - | 19,20,30 | - |
| **30** | 22.69 | 0.99 | d | 3 | 6.5 | - | 19,20,29 | + |

**Supplementary Table S2. Properties of identified *OSC* and *P450* genes from *T. koksaghyz*.** aa, amino acids; bp, base pairs; pI, isoelectric point; kDa, kilodalton; Mw, molecular weight.

|  | **cDNA length [bp]** | **protein size [aa]** | **Mw [kDa]** | **pI** | **accession number** |
| --- | --- | --- | --- | --- | --- |
| **CYP716A263** | 1,422 | 473 | 54.294 | 9.31 | MG646382 |
| **CYP716D60** | 1,440 | 479 | 54.484 | 8.84 | MG646383 |
| **TkLUP** | 2,277 | 758 | 86.705 | 5.77 | MG646375 |
| **TkOSC1** | 2,313 | 770 | 88.543 | 5.70 | MG646376 |
| **TkOSC2** | 2,313 | 770 | 88.714 | 5.69 | MG646377 |
| **TkOSC3** | 2,298 | 765 | 87.908 | 5.69 | MG646378 |
| **TkOSC4** | 2,286 | 761 | 88.548 | 6.17 | MG646379 |
| **TkOSC5** | 2,283 | 760 | 87.260 | 6.25 | MG646380 |
| **TkOSC6** | 2,277 | 758 | 86.946 | 6.05 | MG646381 |

**Supplementary Table S3. Protein sequence data and corresponding accession numbers obtained from GenBank.**

| **Protein** | **Accession number** |
| --- | --- |
| **OSC/LUP proteins** | |
| *A. annua* bAS (AabAS) | ACA13386.1 |
| *A. annua* CAS (AaCAS) | AJE29378.1 |
| *A. annua* LUP (AaLUP) | AJE29379.1 |
| *A. annua* OSC2 (AaOSC2) | AHF22084.1 |
| *A. annua* OSC3 (AaOSC3) | AJE29380.1 |
| *A. thaliana* BARS (AtBARS) | NP_193272.1 |
| *A. thaliana* bAS (AtbAS) | NP_178016.2 |
| *A. thaliana* CAMS (AtCAMS) | NP_683508.1 |
| *A. thaliana* CAS (AtCAS) | NP_178722.1 |
| *A. thaliana* LSS (AtLSS) | NP_190099.3 |
| *A. thaliana* LUP1 (AtLUP1) | NP_178018.1 |
| *A. thaliana* LUP2 (AtLUP2) | NP_178017.2 |
| *A. thaliana* MRN (AtMRN) | NP_199074.1 |
| *A. thaliana* THA (AtTHA) | NP_001078733.1 |
| *E. tirucalli* bAS (EtbAS) | BAE43642.1 |
| *M. truncatula* bAS (MtbAS) | XP_003604121.1 |
| *O. europaea* LUP (OeLUP) | BAA86930.1 |
| *O. europaea* OSC (OeOSC) | BAF63702.1 |
| *P. ginseng*  bAS1 (PgbAS1) | O82140.1 |
| *P. ginseng*  bAS2 (PgbAS2) | O82146.1 |
| *P. ginseng* CAS (PgCAS) | O82139.1 |
| *S. lycopersicum* bAS (SlbAS) | NP_001234604.1 |
| *S. lycopersicum* LUP (SlLUP) | XP_004243674.1 |
| *S. lycopersicum* OSC (SlOSC) | NP_001234597.1 |
| *T. officinale* LUP (ToLUP) | BAA86932.1 |
| *T. officinale* OSC (ToOSC) | BAA86933.1 |
| *T. koksaghyz* LUP1 (TkLUP1) | MG646375 |
| *T. koksaghyz* OSC1 (TkOSC1) | MG646376 |
| *T. koksaghyz* OSC2 (TkOSC2) | MG646377 |
| *T. koksaghyz* OSC3 (TkOSC3) | MG646378 |
| *T. koksaghyz* OSC4 (TkOSC4) | MG646379 |
| *T. koksaghyz* OSC5 (TkOSC5) | MG646380 |
| *T. koksaghyz* OSC6 (TkOSC6) | MG646381 |
| **P450 proteins** | |
| *A. annua* CYP716A14v2 | AHF22083.1 |
| *A. annua* CYP716D22 | AHF22082.1 |
| *A. thaliana* CYP705A1 | NP_193268.3 |
| *A. thaliana* CYP708A2 | NP_851153.1 |
| *A. thaliana* CYP71A16 | NP_199073.1 |
| *A. thaliana* CYP716A1 | NP_198460.1 |
| *A. thaliana* CYP716A2 | NP_198463.1 |
| *B. falcatum* CYP716Y1 | AHF45909.1 |
| *B. platyphylla* CYP716A180 | AHL46848.1 |
| *C. quinoa* CYP716A78 | ANY30853.1 |
| *C. quinoa* CYP716A79 | ANY30854.1 |
| *C. quinoa* CYP716AB1 | ANY30855.1 |
| *C. roseus* CYP716A154 | AEX07772.1 |
| *G. biloba* CYP716B | AHF49536.1 |
| *G. uralensis* CYP88D6 | B5BSX1.1 |
| *M. truncatula* CYP716A12 | CBN88268.1 |
| *M. truncatula* CYP72A67 | XP_013462851.1 |
| *P. ginseng* CYP716A52v2 | I7C6E8.1 |
| *P. grandiflorus* CYP716A141 | AOG74838.1 |
| *P. trichocarpa* CYP716A3 | XP_002324668.2 |
| *P. trichocarpa* CYP716A6 | XP_002325241.2 |
| *P. trichocarpa* CYP716A8 | XP_002309057.1 |
| *S. lycopersicum* CYP716A44 | XP_004239296.1 |
| *S. lycopersicum* CYP716A46 | XP_004243906.1 |
| *S. rebaudiana* CYP716D4 | ABD60225.1 |
| *S. tuberosum* CYP716A13 | XP_006338129.1 |
| *T. koksaghyz* CYP716A263 | MG646382 |
| *T. koksaghyz* CYP716D60 | MG646383 |
| *V. vinifera* CYP716A15 | NP_001268115.1 |
| *V. vinifera* CYP716A17 | NP_001268076.1 |
| *V. vinifera* CYP716A19 | XP_002280969.1 |
| *V. vinifera* CYP716A20 | XP_002264643.4 |

**Supplementary Table S4. Sequences of oligonucleotides used for cloning and qRT-PCR.**

| **Oligo** | **Sequence (5'→3')** |
| --- | --- |
| **full-length cDNA isolation** | |
| CYP716A263-fwd | ATGGCTATGTTCTATGCTT |
| CYP716A263-rev | TTAAAGTTTGTGAGG |
| CYP716D60-fwd | ATGATTCTATTTGTAGC |
| CYP716D60-fwd | TCAAACATGATGAGG |
| TkLUP-fwd | ATGTGGAAGCTGAAAATAGC |
| TkLUP-rev | TCATATATTTTGAACAATACG |
| TkOSC1-fwd | ATGTGGAAGCTGAGAATAGGTG |
| TkOSC1-rev | ttaggtttcttgttttgctaac |
| TkOSC2-fwd | ATGTGGAAGCTGAGAATAGGTG |
| TkOSC2-rev | ttaggtttcttgttttgctaac |
| TkOSC3-fwd | ATGTGGGAGTTAAAGATAGC |
| TkOSC3-rev | TTAATTTTGAATACCCTTTT |
| TkOSC4-fwd | ATGTGGAGGTTAAGAATCGG |
| TkOSC4-rev | TTAGATGATTTTAGACACCC |
| TkOSC5-fwd | ATGTGGAAATTAAAGATAG |
| TkOSC5-rev | TTATTTTTTACACGTGGAC |
| TkOSC6-fwd | ATGTGGAAATTAAAGATAG |
| TkOSC6-rev | CTAGATCCCTTTCAGCACATG |
| **cloning procedures** | |
| AtR2-fwd-NotI | AAAGCGGCCGCATGGGATCCTCTTCTTC |
| AtR2-rev-SpeI | AAAACTAGTTTACCATACATCTCTAAG |
| CYP716A263-fwd-BamHI | AAAGGATCCATGGCTATGTTCTATGCTT |
| CYP716A263-fwd-NcoI | AAACCATGGCTATGTTCTATGCTT |
| CYP716A263-rev-XhoI | AAACTCGAGTTAAAGTTTGTGAGGATAAAG |
| CYP716A263-rev-XmaI | AAACCCGGGTTAAAGTTTGTGAGG |
| CYP716D60-fwd-BamHI | AAAGGATCCATGATTCTATTTGTAGC |
| CYP716D60-rev-XhoI | AAACTCGAGTCAAACATGATGAGG |
| pESC-URA-fwd | CTATAAAAAAATAAATAGGGAC |
| pESC-URA-rev | GGCTGCGCAACTGTTGG |
| Tkhmgrc1-fwd-PciI | AAAACATGTCAGAGAACGAGGAGATCGTGAAGC |
| Tkhmgrc1-rev-XhoI | AAACTCGAGCTAATTAGTGGCTAATTTG |
| TkLUP-fwd-SalI | AAAGTCGACTATGTGGAAGCTGAAAATAGC |
| TkLUP-fwd-Sc-SalI | AAAGTCGACTAAAAAAATGTGGAAGCTGAAAATAGC |
| TkLUP-rev-Sc-Nterm | GTGGTTGTTGGTGGTGGT |
| TkLUP-rev-XhoI | AAACTCGAGTCATATATTTTGAACAATACG |
| TkOSC1-fwd-NcoI | aaaccatggaatggaagctgagaataggtg |
| TkOSC1-fwd-Sc-Cterm | ACCACCACCAACAACCACGTGGGGAGACAAACATG |
| TkOSC1-rev-XhoI | aaactcgcgttaggtttcttgttttgctaac |
| TkOSC2-fwd-NcoI | aaaccatggaatggaagctgagaataggtg |
| TkOSC2-rev-XhoI | aaactcgcgttaggtttcttgttttgctaac |
| TkOSC5-fwd-SalI | AAAGTCGACAATGTGGAAATTAAAGATAGC |
| TkOSC5-rev-XhoI | AAACTCGAGTTATTTTTTACACGTGGAC |
| TkOSC6-fwd-SalI | AAAGTCGACAATGTGGAAATTAAAGATAGC |
| TkOSC6-fwd-Sc-KpnI | AAAGGTACCAAAAAAATGTGGAAATTAAAGATAG |
| TkOSC6-rev-XhoI | AAACTCGAGCTAGATCCCTTTCAGCACATG |
| **qRT-PCR** | |
| CYP716A263-fw-realtime | GCACGCACCTTCATTGGAAA |
| CYP716A263-rv-realtime | GCAGTGTCGGCCATGTATTT |
| CYP716D60-fw-realtime | TGCAGGTTGGGAAGGAAAAC |
| CYP716D60-rv-realtime | GCCACAGAGAACTGCGATAC |
| TkEF1alpha-fw-realtime | CGAGAGATTCGAGAAGGAAGC |
| TkEF1alpha-rv-realtime | CTGTGCAGTAGTACTTGGTGG |
| TkLUP-fw-realtime | GCTGACCACCACCAACAACCAC |
| TkLUP-rv-realtime | AGCACGTTCCTCTTCGGTTCCAG |
| TkOSC1-fw-realtime | ACTCCTCCCTTGATAATTGCCC |
| TkOSC1-rv-realtime | TTGTGCTTCTGCCTGATATATAGAAC |
| TkOSC2-fw-realtime | CCGGTGAGAAGGTGGAAGTT |
| TkOSC2-rv-realtime | GGAACCGGTACCTCCCAAAC |
| TkOSC3-fw-realtime | TCCATCCAAACCACAGAAAAG |
| TkOSC3-rv-realtime | ATGAAGCATACTCCCCAATAAC |
| TkOSC4-fw-realtime | CCGACAATTCGTAAGGCCACTG |
| TkOSC4-rv-realtime | TGTTTGCACCACGTTCGACC |
| TkOSC5-fw-realtime | GAAACACAACTAGAAGATGGCGGT |
| TkOSC5-rv-realtime | CATAGCCCATGAAGTGTGCACT |
| TkOSC6-fw-realtime | GGTCATAGCACCATGTTTGGG |
| TkOSC6-rv-realtime | GGTGACTGAGCCATGATCCAGG |
| TkRP-fw-realtime | CGTCGATCTCAAGGATGTTGTC |
| TkRP-rv-realtime | GGAGCTTTGAGAAGAACCAACG |

**Supplementary Table S5. Primer efficiency and amplification factors for cDNA obtained from *T. koksaghyz* mRNA.** The values were calculated using the Bio-Rad CFX Manager v3.1 software (Bio-Rad Laboratories Inc., Hercules, CA, USA) and the qPCR primer efficiency calculator provided by Thermo Fisher Scientific (http://www.thermoscientificbio.com/webtools/qpcrefficiency/).

| **Oligo pair** | **Efficiency** | **Amplification factor (66°C)** |
| --- | --- | --- |
| CYP716A263-realtime | 107.62% | 2.08 |
| CYP716D60-realtime | 102.83% | 2.03 |
| TkLUP-realtime | 99.17% | 1.99 |
| TkOSC1-realtime | 100.16% | 2.00 |
| TkOSC2-realtime | 101.87% | 2.02 |
| TkOSC3-realtime | 109.67% | 2.10 |
| TkOSC4-realtime | 108.54% | 2.09 |
| TkOSC5-realtime | 106.95% | 2.07 |
| TkOSC6-realtime | 94.39% | 1.94 |
| TkEF1alpha-realtime | 104.48% | 2.04 |
| TkRP-realtime | 105.44% | 2.05 |
